# Corn Straw Biochar–OPC Composites for Sustainable Treatment of Water-Rich Dredged Clay: Internal Curing Mechanisms, Pore-Structure Evolution, and Mechanical–Environmental Performance

**DOI:** 10.3390/ma19163369

**Published:** 2026-08-07

**Authors:** Wenrui Xu, Zichen Zhang, Zhicheng Dong, Hao Li, Gaofeng Xie

**Affiliations:** 1 Department of School of Architecture & Civil Engineering, Liaocheng University, Liaocheng 252000, China; 13954133091@163.com (W.X.); dzc16505440879@163.com (Z.D.); 2 Department of Shandong Second Water Conservancy Engineering Bureau Co., Ltd., Jining 272100, China

**Keywords:** sustainable sediment management, high-water-content dredged clay, corn straw biochar, OPC replacement, pore-structure regulation, life-cycle assessment

## Abstract

**Highlights:**

- CSB700-30 showed the best performance, with the highest water absorption of 1476.10% and a 28 d UCS of 136–137 kPa.
- CSB improved clay strength through water regulation, pore filling, and interfacial bonding, with microstructural evidence suggesting the formation of Ca–Si-rich hydration products.
- CSB700-30 achieved the best balance between strength improvement and carbon reduction.
- CSB–OPC offers a low-carbon approach for dredged clay stabilization, but further validation using natural dredged clays is needed.

**Abstract:**

High-water-content dredged clay is a water-rich sediment that requires sustainable, resource-oriented treatment, but conventional ordinary Portland cement (OPC) stabilization is hindered by excessive water-to-binder ratios, suppressed hydration, and high carbon emissions. This study investigated corn straw biochar (CSB) as a partial OPC substitute for the low-carbon stabilization of kaolin-based simulated dredged clay. CSB prepared at 200–1000 °C under different holding times was characterized by water absorption, thermogravimetry, and SEM. Clay specimens with an initial water content of 80% (1.5 *w*_L_) were prepared using 5–12% total binder, CSB:OPC ratios of 5:5–8:2, and curing ages of 7 and 28 d, and were evaluated by UCS, pH, SEM/ESEM, EDS, and life-cycle-based mechanical–environmental assessment. CSB developed honeycomb-like interconnected pores and a lamellar carbon skeleton, reaching 1476.10% water absorption at 700 °C for 30 min. The optimum CSB–OPC specimen reached approximately 136–137 kPa at 28 d, exceeding the OPC-only control of about 102 kPa. Strength improvement was associated with alkalinity maintenance, Ca–Si-rich hydration products, pore filling, and CSB–clay interfacial adsorption, embedding, and encapsulation. Properly carbonized CSB shows potential as an internal-curing, low-carbon co-binder for simulated dredged clay, but its field applicability requires further validation.

## 1. Introduction

With the continuous expansion of port construction, channel dredging, coastal reclamation, and waterway maintenance projects, large quantities of dredged sediments are generated worldwide. High-water-content dredged clay is one of the most difficult fractions to manage because it usually contains abundant free water, exhibits a high void ratio and compressibility, and has low bearing capacity and poor structural stability [1,2,3,4]. These properties restrict its direct reuse in roadbed filling, foundation improvement, and land reclamation [5,6]. In addition, long-term stockpiling or uncontrolled disposal of untreated dredged clay may occupy land resources and induce secondary environmental risks, including heavy-metal migration, groundwater contamination, and ecological degradation [1,7]. Therefore, developing efficient, low-carbon, and resource-oriented treatment strategies for high-water-content dredged clay is important not only for geotechnical engineering but also for sustainable water treatment and sediment management [3,8,9,10,11,12,13,14,15].

Several treatment methods have been developed for water-rich dredged clay, including mechanical dewatering, vacuum preloading, thermal treatment, and chemical solidification/stabilization. Mechanical dewatering can remove free and capillary water through filtration or centrifugation, but its efficiency is often limited for fine-grained clay with poor drainage pathways, and large treatment areas or long processing times are usually required [16]. Vacuum preloading can accelerate consolidation by applying negative pressure and promoting pore-water discharge; however, its treatment rate remains relatively slow for clay with high initial water content and low permeability [17]. Thermal treatment can rapidly reduce water content through evaporation, but it is commonly associated with high energy consumption, potential pollution risks, and limited large-scale applicability [18]. These limitations indicate that the sustainable treatment of high-water-content dredged clay still requires material-based strategies that can simultaneously regulate water, improve strength, and reduce environmental burden.

Cement-based stabilization has been widely used because of its mature construction technology, reliable strength enhancement, and strong engineering adaptability [19]. Nevertheless, the use of ordinary Portland cement (OPC) is accompanied by high energy consumption and considerable carbon emissions. More importantly, under high-water-content conditions, the excessive water-to-binder ratio weakens cement hydration by diluting ions, increasing ion diffusion distances, and inhibiting the formation of calcium silicate hydrate (C–S–H) gels [20,21]. As a result, stabilized clay may retain high porosity, low strength, and increased susceptibility to shrinkage cracking. Therefore, reducing OPC consumption while maintaining or improving the stabilization efficiency of water-rich dredged clay has become a key challenge in the development of low-carbon treatment technologies.

Biochar has attracted increasing attention as a supplementary material for cement-stabilized clay because of its porous structure, high specific surface area, surface functional groups, and potential carbon-storage benefits [22,23,24]. However, the effectiveness of biochar in cementitious stabilization systems remains dependent on feedstock type, carbonization temperature, residence time, pore structure, and chemical composition. Some studies emphasize that biochar can act as a water reservoir, nucleation substrate, and pore-filling modifier, thereby promoting internal curing and microstructural densification. Other studies indicate that inappropriate biochar may introduce excessive inert ash, residual organic components, or weak interfacial zones, which can reduce hydration efficiency and compromise mechanical performance [25,26]. These diverging observations suggest that biochar cannot be treated as a universally beneficial additive; rather, its stabilization effect must be evaluated in relation to its microstructure, water-regulation capacity, and interaction with cement hydration products. Therefore, corn straw biochar (CSB) was selected in this study not only for agricultural-waste valorization, but also for its potential compatibility with the stabilization requirements of high-water-content dredged clay. After controlled pyrolysis, corn straw can retain a tubular and honeycomb-like carbon skeleton with interconnected pores and surface-active sites. This porous structure is expected to absorb part of the excessive free water, provide water-storage sites during curing, and offer attachment surfaces for clay particles and cement hydration products. Compared with untreated straw, CSB also contains fewer easily degradable organic components that may inhibit cement hydration in an alkaline environment. Therefore, CSB may function not only as a partial OPC substitute, but also as a water-regulating and microstructure-modifying component in CSB–OPC-stabilized clay.

Corn straw is an abundant lignocellulosic agricultural by-product, and its open burning or improper disposal can cause air pollution and waste biomass resources [27,28]. Through pyrolysis, corn straw can be converted into biochar with a stable carbon-rich skeleton, interconnected pores, and abundant surface-active sites within a relatively short carbonization time [29,30,31]. Compared with some ash-rich or highly lignified biochars, corn straw biochar (CSB) may provide a more favorable pore structure for water absorption, internal curing, and interfacial bonding in high-water-content clay. Its use in OPC-stabilized dredged clay could therefore combine agricultural-waste valorization, cement reduction, water regulation, and low-carbon stabilization. However, the relationship between CSB carbonization conditions, water absorption behavior, pore-structure evolution, and mechanical–environmental performance in high-water-content dredged clay remains insufficiently understood.

Existing studies on biochar-based clay improvement have mainly focused on ordinary soft clay, agricultural clay, contaminated clay, or cement-based composites with relatively moderate water contents. Systematic investigations on simulated high-water-content dredged clay are still limited. In particular, the mechanisms by which CSB regulates free water, supports cement hydration, modifies pore structure, and contributes to interfacial cementation among biochar, clay particles, and hydration products have not been fully clarified. This knowledge gap restricts the rational design of biochar–cement composite stabilizers for water-rich dredged sediment treatment.

In this study, CSB was introduced as a partial OPC substitute for the sustainable stabilization of a kaolin-based simulated high-water-content dredged clay. The effects of carbonization temperature and residence time on CSB water absorption, thermal behavior, and microstructure were first characterized. Stabilized clay specimens were then prepared with different binder dosages, CSB:OPC mass ratios, and curing ages. Unconfined compressive strength tests, pH measurements, SEM/ESEM observations, EDS analysis, and life-cycle-based mechanical–environmental assessment were conducted to evaluate the stabilization performance and underlying mechanisms. The main aim was to examine how CSB water-regulation behavior and microstructural modification are associated with strength development and mechanical–environmental performance. The results suggest that properly carbonized CSB may support hydration-related microstructure development, improve CSB–clay interfacial bonding, and provide a more favorable balance between mechanical strength and carbon-emission intensity under controlled laboratory conditions. This work provides a potential low-carbon and resource-oriented material strategy for the sustainable management of high-water-content dredged clay, although further validation using natural dredged clays with different mineral compositions, particle gradations, salinity levels, and organic matter contents is still required.

## 2. Materials and Methods

### 2.1. Materials

A kaolin–water system was used in this study to simulate high-water-content dredged clay. The kaolin was purchased from Shengyun Minerals, Lingshou County, Shijiazhuang City, Hebei Province, China, and its particle size distribution was determined in accordance with ISO 17892-4:2016 [32]. As shown in Table 1, the kaolin had a liquid limit (*w*_L_) of 53.26%, a plastic limit (*w*_P_) of 33.14%, a plasticity index (*I*_P_) of 20.12, a particle density of 2.64 g/cm^3^, a loss on ignition of 12.58%, and fine and coarse particle contents of 86.70% and 13.30%, respectively. Kaolin was selected instead of natural dredged clay to minimize the effects of variability in the composition of undisturbed dredged clay, differences in organic matter content, and salt interference on the curing reaction and the repeatability of strength tests [33,34]. The kaolin–water system was used as a simplified and reproducible model clay to clarify the effects of CSB carbonization conditions and CSB–OPC proportions on hydration-related behavior, water regulation, and strength development. However, natural dredged sediments may contain more complex mineral phases, variable organic matter contents, soluble salts, sulfides, heavy metals, shell fragments, and heterogeneous particle-size fractions, which can affect cement hydration and biochar–clay interactions. Therefore, the present results should be interpreted as laboratory-scale mechanistic evidence obtained from simulated dredged clay rather than direct field validation.

**Figure 1:**
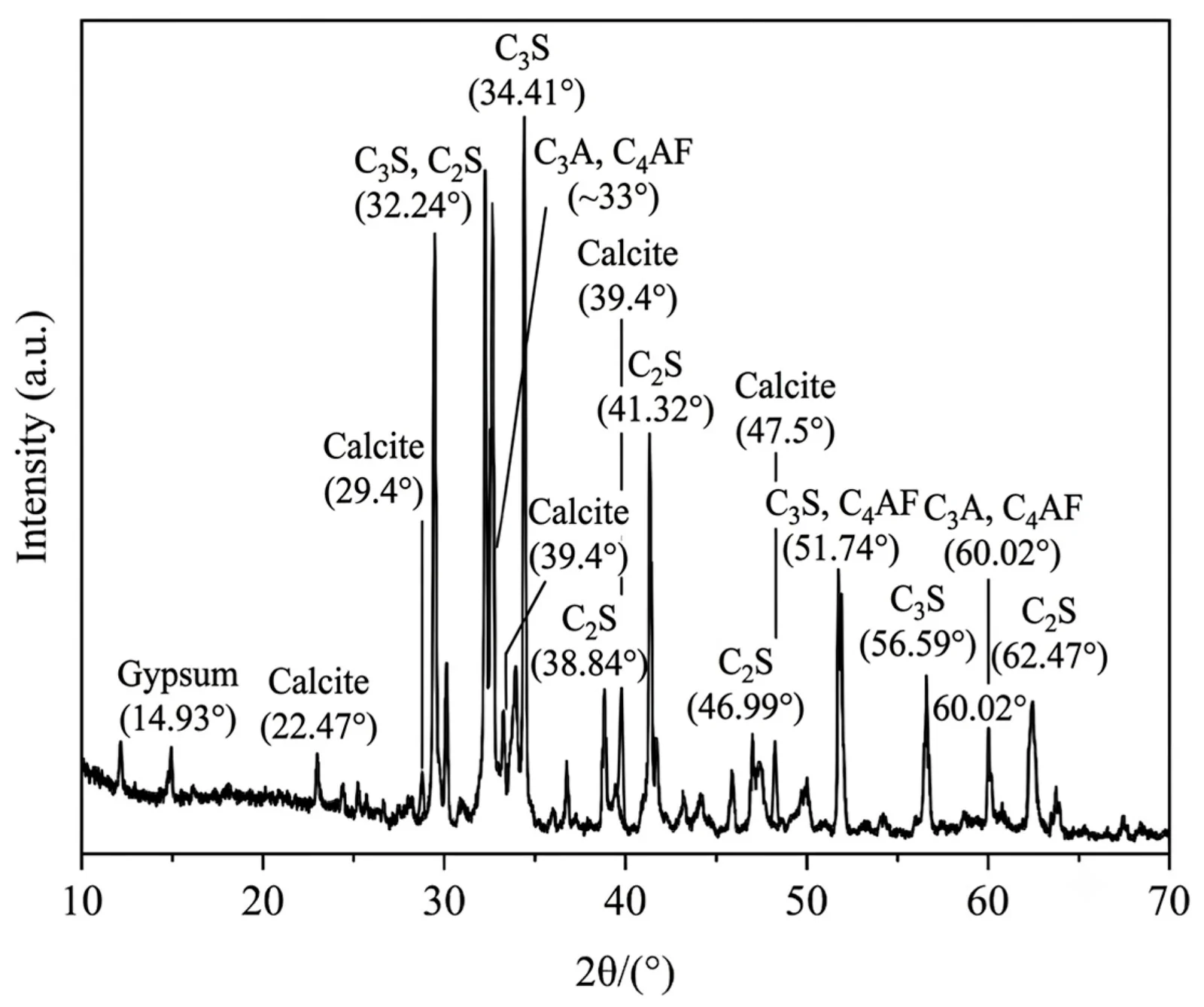
XRD patterns of OPC.

The corn straw used in this study was collected from the residual biomass remaining in the field after corn harvest. After collection, the material was manually sorted to remove leaves, roots, adhering clay, and other visible impurities, and only the stalks were retained as the pyrolysis feedstock. The binder used in this study was ordinary Portland cement (OPC) produced by Anhui Conch Cement Co., Ltd. (Wuhu, China), with a strength grade of P·O 52.5. Its main chemical composition is presented in Table 2. To further characterize the mineralogical composition of the cement, X-ray diffraction (XRD) analysis was performed in accordance with ASTM C1365-18 (2023) [35], and the resulting diffraction pattern is shown in Figure 1.

### 2.2. Preparation and Characterization of Corn Straw Biochar

The preparation procedure for corn straw biochar (CSB) is summarized in Table 3. The collected corn straw was cut into 30–50 mm segments, washed, and oven-dried at 105 °C for 24 h. Approximately 50 g of the dried stalks was then packed into a sealed metal container with an inner diameter of 150 mm and a height of 80 mm. The container was heated at a rate of 10 °C/min under a static oxygen-limited atmosphere. After the target temperature was reached, the sample was held for the specified duration and then allowed to cool naturally to room temperature in the furnace. The resulting carbonized straw was manually ground and sieved through a 2 mm mesh to obtain a uniform CSB powder.

**Figure 2:**
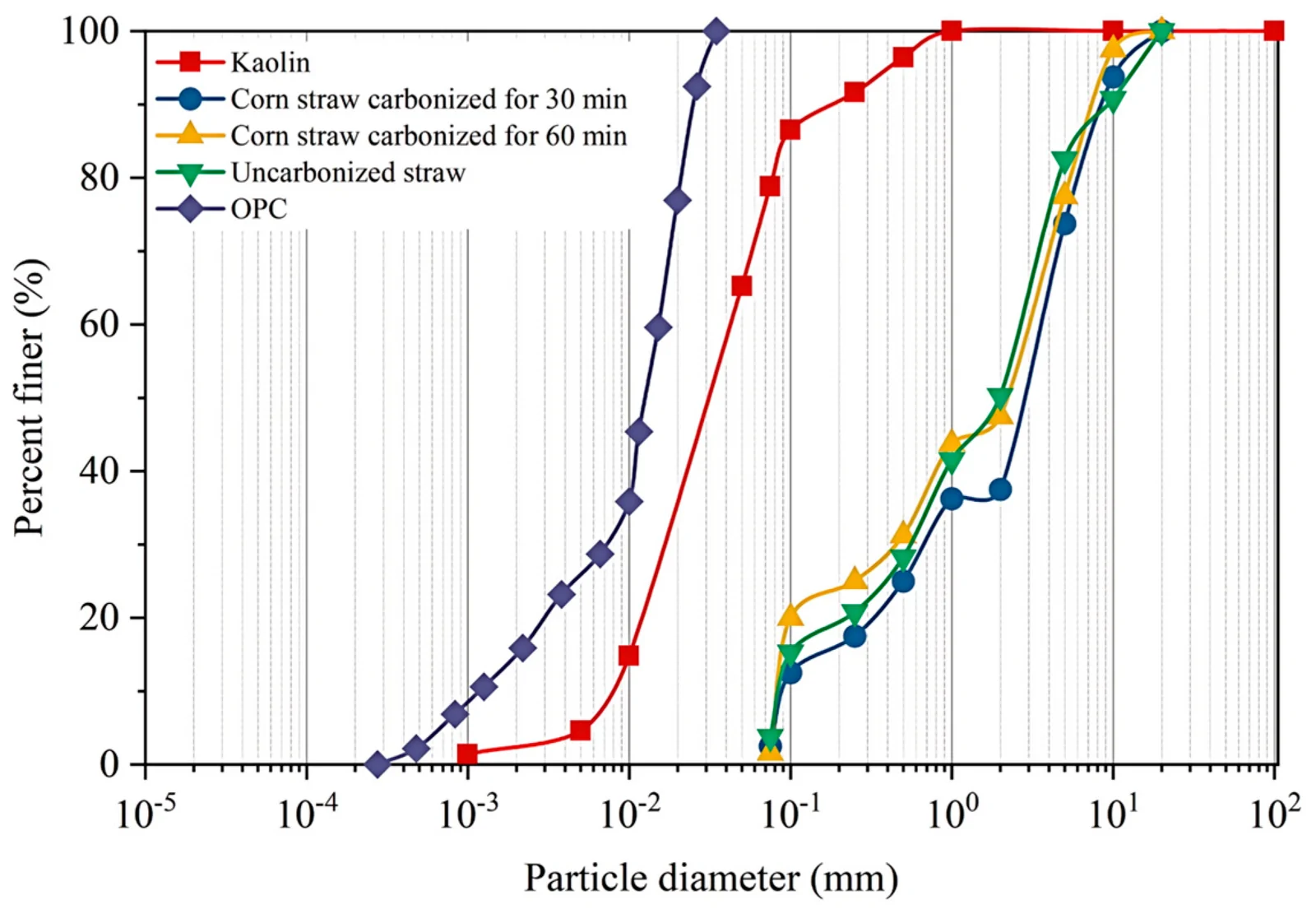
Grain size distribution of the materials.

To analyze the particle size distribution (PSD), corn stover was pyrolyzed at 700 °C under oxygen-limited conditions for residence times of 30 min and 60 min. After cooling to room temperature, each biochar sample was ground for 1 min in a grinder together with an equal mass of raw uncharred corn stover. The PSDs of the resulting CSB particles were then compared with those of the uncharred stover, kaolin, and OPC, as shown in Figure 2.

**Figure 3:**
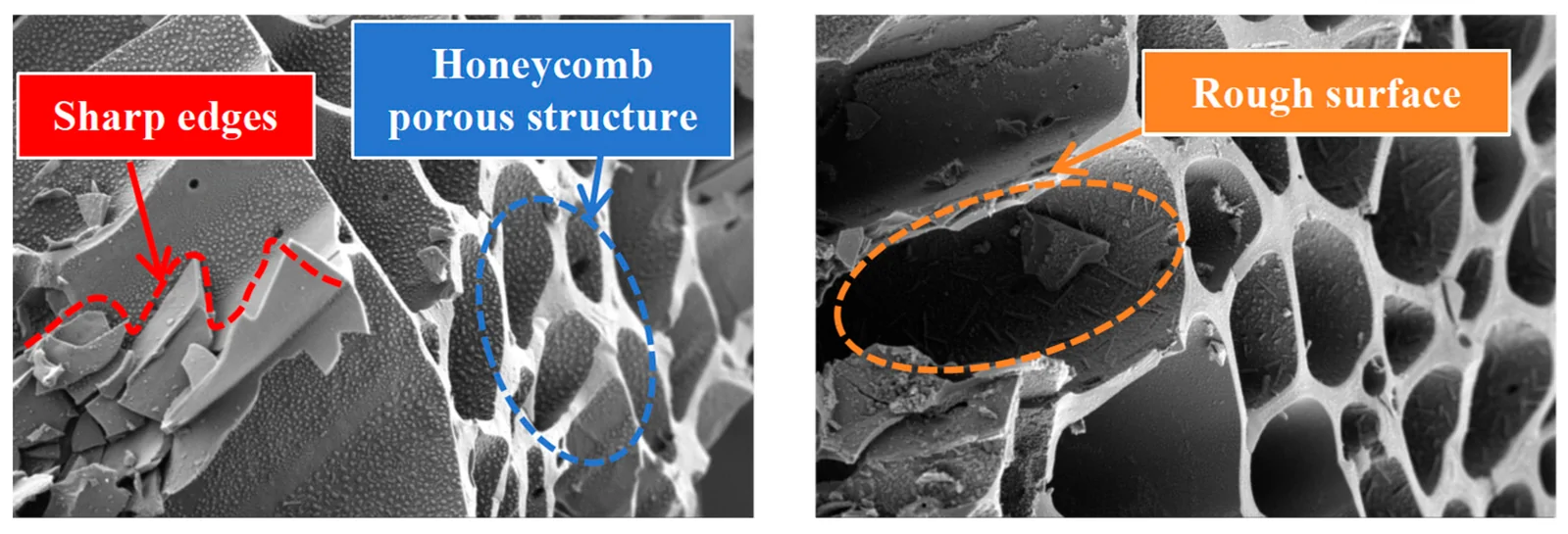
Microstructure of corn straw biochar.

Scanning electron microscopy (SEM) (Carl Zeiss AG, Oberkochen, Germany) was used to observe the morphology of CSB particles prepared at 700 °C. As shown in Figure 3, the biochar retained the original vascular architecture of maize straw, exhibiting a regular honeycomb-like pore network and a lamellar carbon skeleton. The pore walls appeared rough and were coated with numerous fine particulate residues, while sharp jagged edges and flake-like fragments were observed throughout the CSB particles.

**Figure 4:**
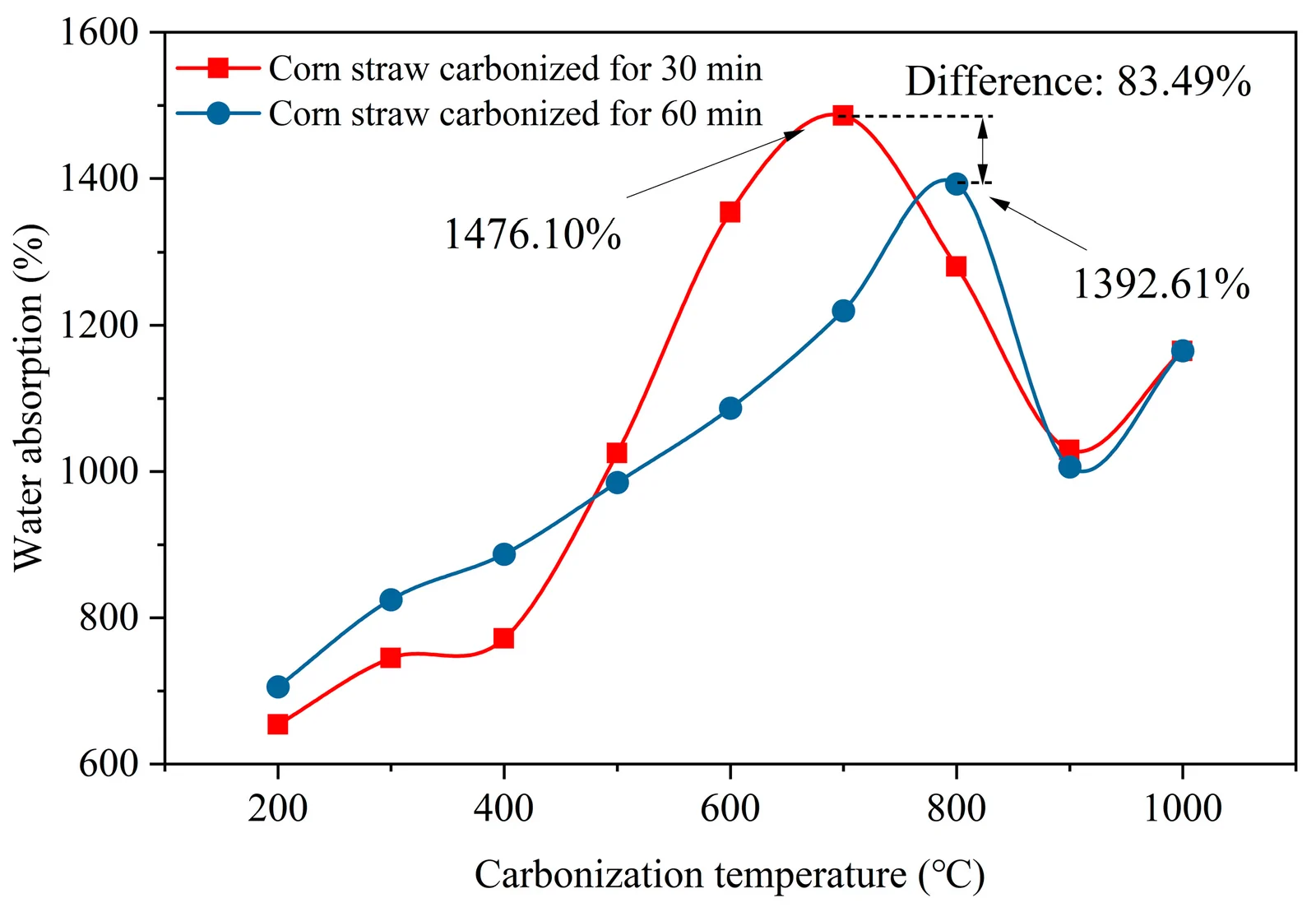
Water absorption rate of corn straw biochar.

The water absorption capacity of the CSB samples was measured using a modified soaking method in accordance with ISO 62:2008 [36]. Each test was conducted in triplicate, and the average values were reported. Figure 4 presents the water absorption of CSB carbonized at different temperatures. Notably, the sample carbonized at 700 °C for 30 min exhibited the highest water absorption, reaching 1476.10%.

**Figure 5:**
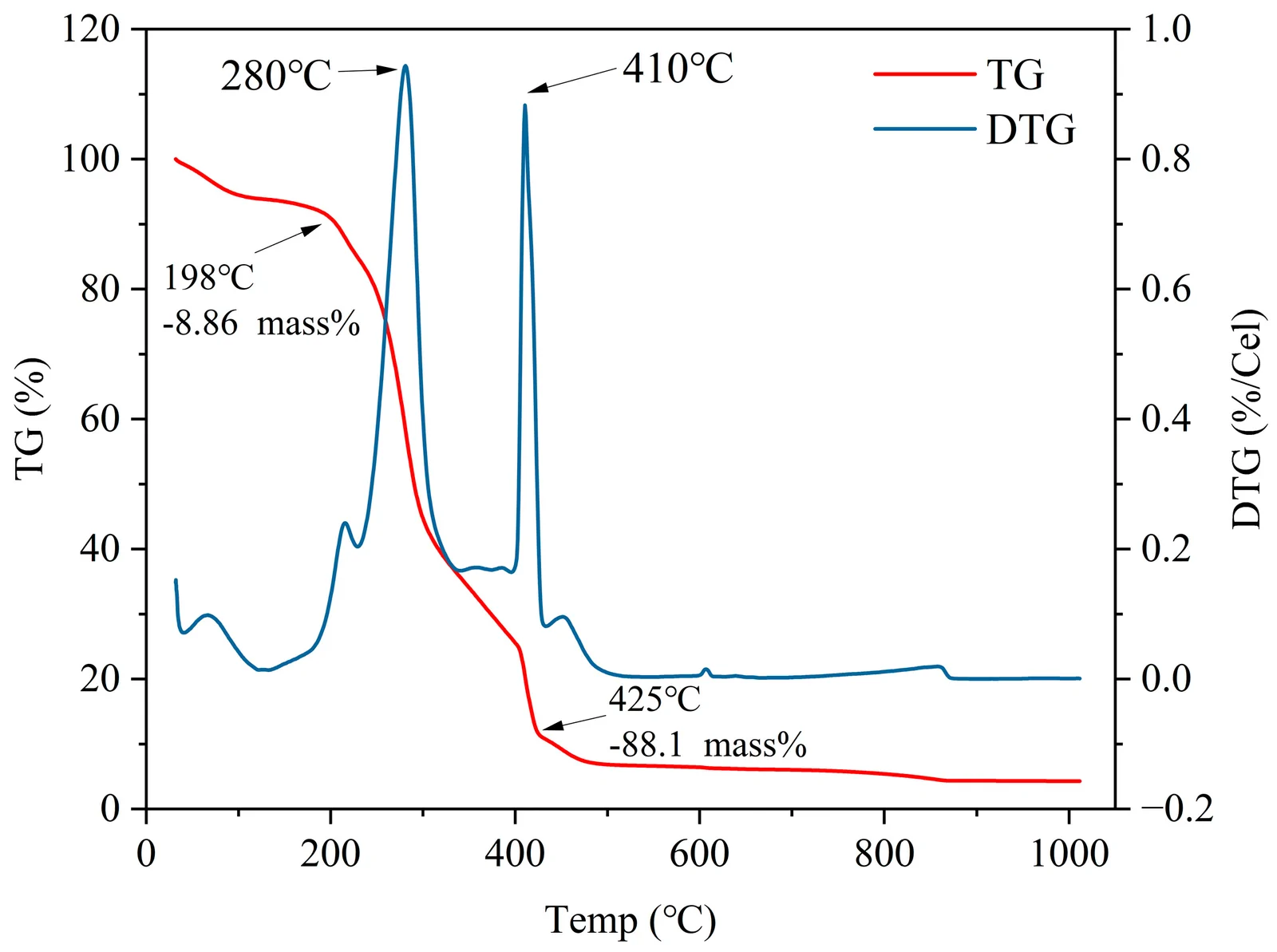
TG–DTG curves of carbonized corn straw.

Thermogravimetric analysis (TGA) was performed according to ISO 11358-1:2022 [37] to evaluate the thermal stability of CSB. The sample was heated from room temperature to 1000 °C at a rate of 10 °C/min under an air atmosphere. The resulting TG curve, shown in Figure 5, indicates a major weight-loss event below 425 °C. The derivative thermogravimetric (DTG) curve exhibits two distinct peaks near 280 °C and 410 °C, corresponding to the decomposition of hemicellulose and cellulose, respectively [38,39]. Above 425 °C, the TG curve levels off, indicating that most thermally labile components had been eliminated and that a more stable carbonaceous framework remained. These carbonized products, which maintain structural stability at high temperatures, exhibit good chemical stability and can retain their integrity and water absorption capacity when incorporated into stabilized clay or cement systems [40,41]. This provides a scientific basis for their use as low-carbon stabilization materials.

In the present study, CSB was characterized in terms of particle-size distribution, water absorption, thermal behavior, and morphology, because the main objective was to examine its water-regulation role in high-water-content clay. Additional parameters commonly used for biochar characterization, such as BET specific surface area, pore-size distribution, elemental composition, ash content, and surface functional groups, were not measured. Therefore, the interpretation of the effects of pyrolysis conditions is mainly based on the measured water absorption, SEM morphology, TG/DTG behavior, and the corresponding mechanical response of the stabilized clay.

### 2.3. Mix Proportion Design

To investigate the synergistic stabilization effect of the CSB–OPC composite system on high-water-content dredged clay, a series of specimens were prepared and subjected to unconfined compressive strength (UCS) tests. The key variables considered in the mix design included carbonization temperature, carbonization time, total stabilizer dosage, and the CSB-to-OPC mass ratio. OPC and CSB were employed as stabilizers, as summarized in Table 4 and Table 5. The total stabilizer content was calculated based on the dry mass of kaolin and was set at four levels: 5%, 7%, 10%, and 12%.

The specimens were divided into three groups: (1) OPC-stabilized high-water-content dredged clay, (2) raw corn straw–OPC composite-stabilized clay, and (3) CSB–OPC composite-stabilized clay. For the first stage of testing, CSB was prepared by pyrolyzing corn straw at 700 °C for either 30 min or 60 min. To investigate the interaction between straw or CSB and OPC, four mass ratios of straw or CSB to OPC were tested: 5:5, 6:4, 7:3, and 8:2. All specimens in this stage were cured for 7 d and 28 d to evaluate early-age and later-age strength development.

In the second stage, the effects of carbonization temperature and residence time were further examined. CSB samples were prepared at carbonization temperatures of 500, 600, 700, and 800 °C, with residence times of 30 min and 60 min. The same total stabilizer dosages and CSB mass ratios were used, and the specimens were cured for 28 d before testing.

### 2.4. Sample Preparation and Curing

**Figure 6:**
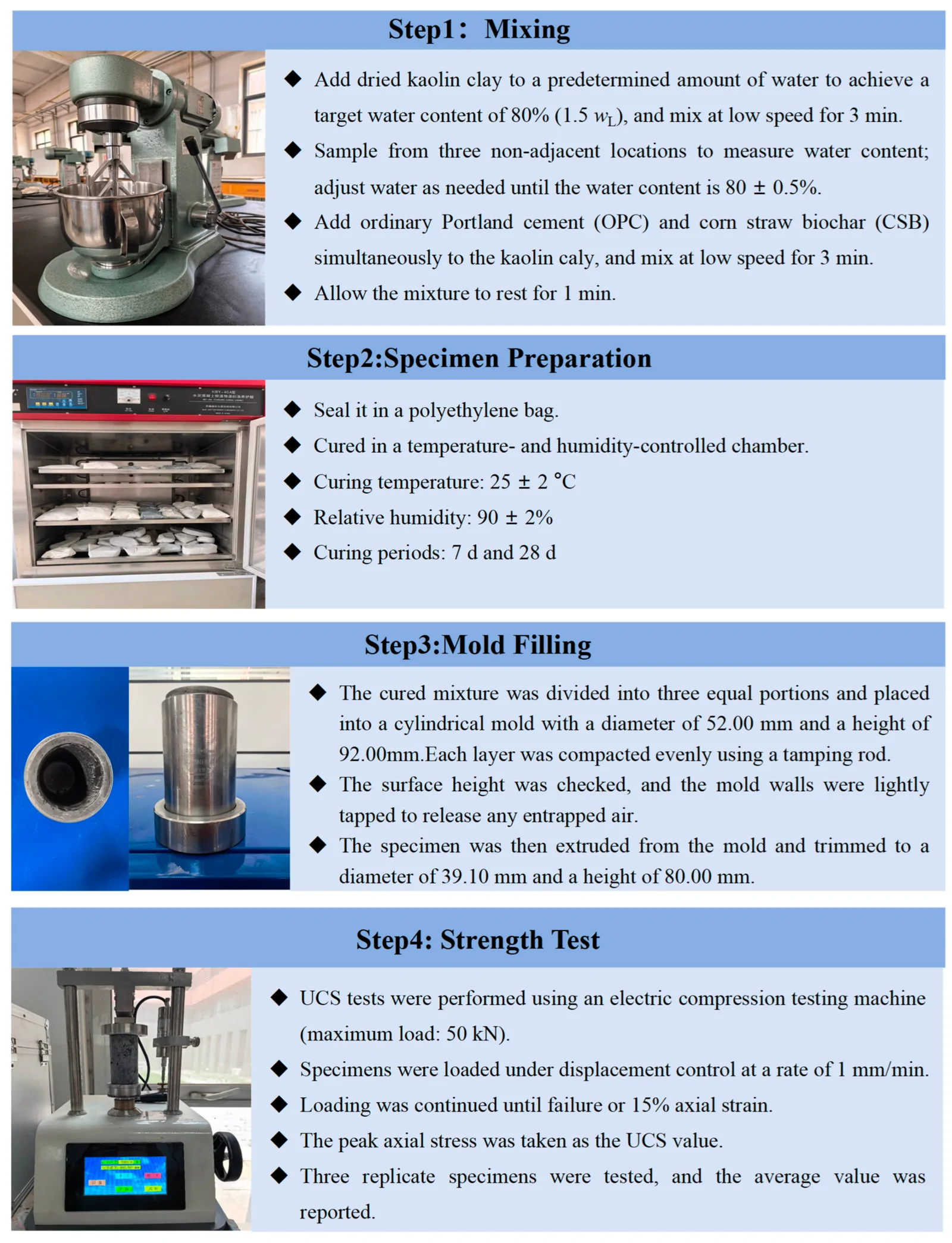
Specimen Preparation Process.

The specimen preparation procedure is shown in Figure 6. Dry kaolin clay was first mixed with a predetermined amount of water to prepare a slurry with a target water content of 80%, corresponding to 1.5 wL. The mixture was mechanically stirred at a low speed of 65 ± 5 rpm for 3 min. Samples were then taken from three non-adjacent locations to measure the water content, and the amount of water was adjusted as needed until the water content was controlled at 80 ± 0.5%.

Subsequently, OPC and CSB were added simultaneously to the kaolin slurry, followed by another 3 min of low-speed mixing and 1 min of resting. After mixing, the specimens were sealed with polyethylene film to prevent moisture evaporation and absorption. All specimens were placed in a standard curing chamber on the day of preparation and cured at a constant temperature of 25 ± 2 °C and a relative humidity of 90 ± 2%. The curing periods were 7 d and 28 d before subsequent testing.

Specimen preparation was performed in accordance with ASTM D2166/D2166M [42]. After the clay–water–OPC–CSB slurry was mixed, its pH was measured using a calibrated digital pH meter. The resulting mixture was then evenly divided into three equal portions and placed sequentially in a cylindrical mold with an inner diameter of 52.00 mm and a height of 92.00 mm. The mold was filled in three layers, and each layer was uniformly tamped with a rod to remove entrapped air and ensure good interlayer bonding.

After all three layers had been placed, the mold was subjected to static compaction using a hydraulic press until the specimen height was reduced to 80.00 mm. The mold was then unloaded, and the specimen was demolded to obtain a cylindrical sample with a diameter of 39.10 mm and a height of 80.00 mm.

After curing, the mechanical performance of the stabilized clay was evaluated using the UCS test. Each specimen was placed at the center of the loading platform of a computerized UCS testing machine and subjected to continuous axial loading under displacement control at a rate of 1.0 mm/min until failure occurred or an axial strain of 15% was reached. During the test, load and displacement data were continuously recorded, and the axial stress was calculated based on the specimen cross-sectional area. To ensure reliability, three replicate specimens were prepared for each mix proportion and tested under identical conditions. The average compressive strength of the three replicate specimens was reported as the UCS for each group.

### 2.5. Mechanical–Environmental Assessment

A preliminary life-cycle-based mechanical–environmental assessment was conducted to compare the carbon-emission efficiency of OPC-only and CSB–OPC-stabilized clay. The system boundary included OPC production and CSB preparation, including corn straw drying, pyrolysis, grinding, and sieving. Transportation, field mixing, curing, construction equipment, and end-of-life stages were not included because this study focused on laboratory-scale material comparison rather than full field implementation.

The global warming potential (GWP) of each mixture was calculated by summing the carbon emissions associated with OPC production and CSB preparation:

(1) $$GWP=m_{OPC}\times {EF}_{OPC}+m_{CSB}\times {EF}_{CSB}$$

where m_OPC_ and m_CSB_ represent the masses of OPC and CSB used in each mixture, respectively, and EF_OPC_ and EF_CSB_ denote the corresponding emission factors. The carbon emission intensity (CI) was then calculated as follows:

(2) $$\mathrm{CI}\;=\;\frac{GWP}{UCS}$$

where CI denotes carbon emission intensity, GWP is expressed in g CO_2_-eq, and UCS is the 28 d unconfined compressive strength expressed in kPa. A lower CI indicates that less carbon emission is required to achieve a unit strength level. The emission factors and assumptions used in this study are summarized in Table 6.

### 2.6. Statistical Analysis

All UCS and water-absorption tests were conducted in triplicate, and the results are reported as mean ± standard deviation. One-way analysis of variance (ANOVA) was used to evaluate significant differences among selected mixtures. When significant differences were detected, Tukey’s post hoc test was applied at a significance level of *p* < 0.05. For the mechanical–environmental assessment, CI values were calculated using the mean UCS values, and the uncertainty was estimated from the standard deviation of UCS.

## 3. Results

### 3.1. Strength Development and pH Characteristics of Stabilized Clay

**Figure 7:**
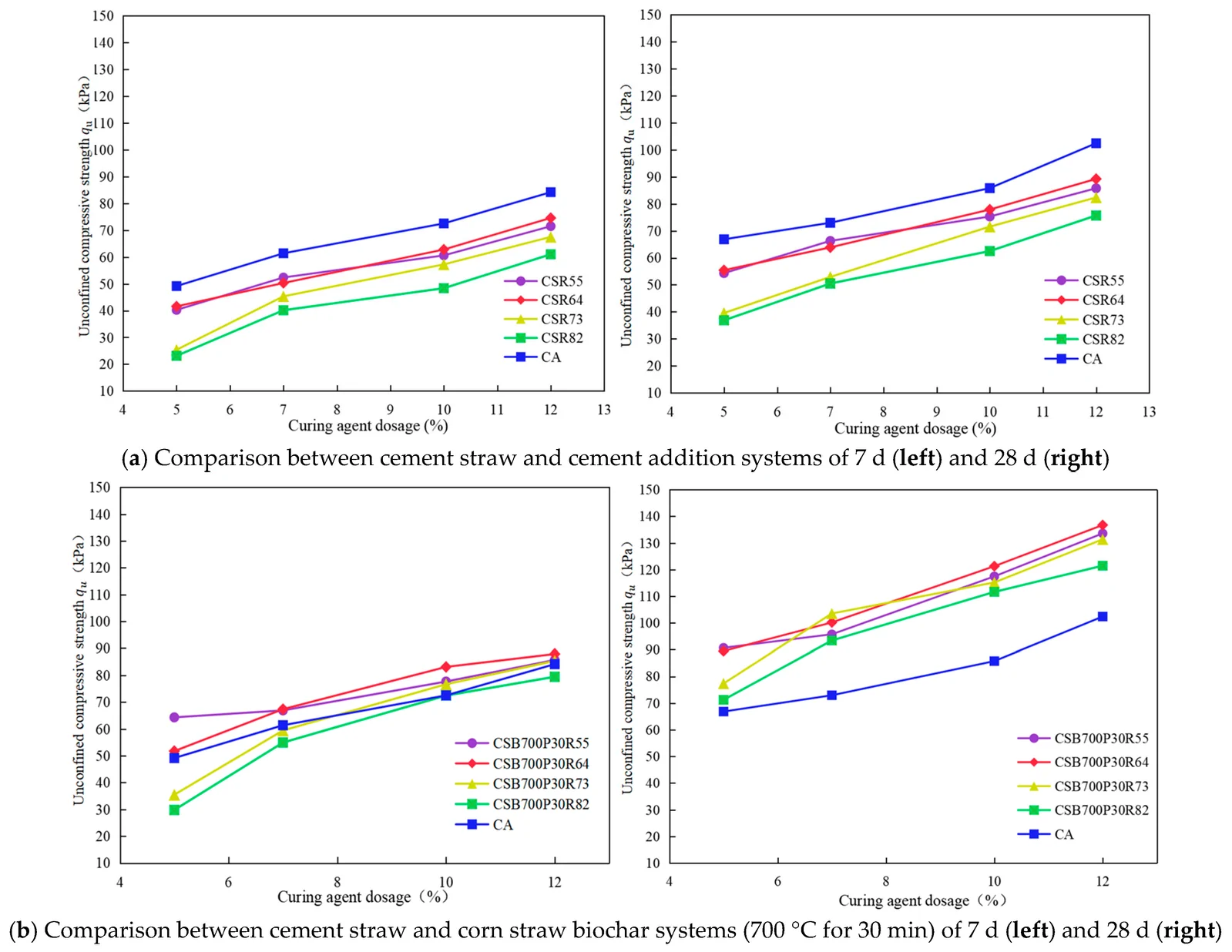

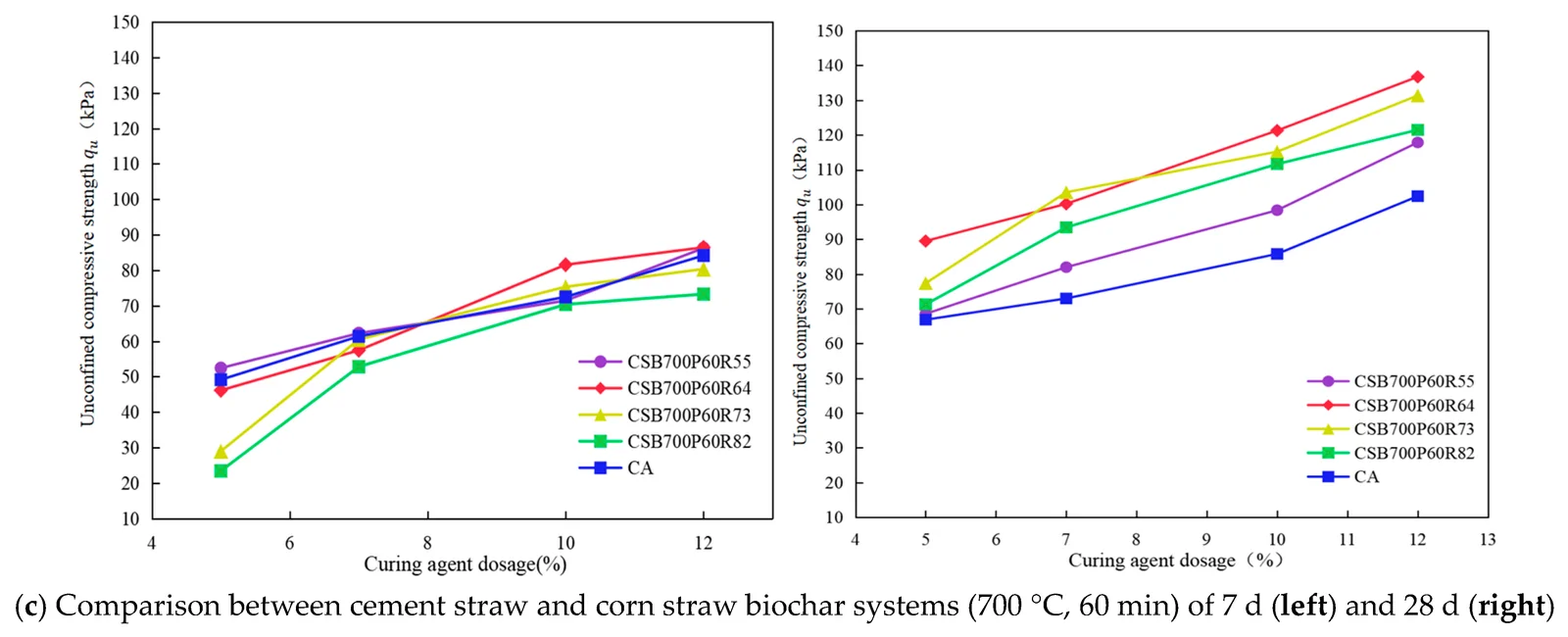
UCS of stabilized clays at different curing agent dosages after 7 d and 28 d of curing.

Figure 7 shows the unconfined compressive strength (UCS) results of samples with varying binder dosages at curing ages of 7 and 28 days. Previous studies have shown that binder dosage and moisture content are two critical factors controlling the consolidation effectiveness of clay [43]. The experimental results indicate that the strength of all sample groups increases with increasing binder dosage, and that the 28-day strength is generally higher than that at 7 days, suggesting that with prolonged curing, cementitious products continue to form and promote the densification of the clay structure.

As shown in Figure 7a, compared to the OPC-stabilized clay, under the same binder dosage the CS–OPC samples (CS group) containing powdered dried straw exhibit a continuous decrease in strength with increasing CS content. This may be due to the organic components in the straw, such as cellulose, hemicellulose, and lignin, releasing organic acids, carboxyl groups, and other substances in an alkaline environment; these substances consume some hydroxide (OH^−^) ions, thereby inhibiting the hydration of OPC [44]. At the same time, the interface bonding between the straw and the cementitious matrix is relatively weak and tends to form structural weak zones, which further weakens the stabilization effect [45].

Compared with the consolidation effect of untreated (non-carbonized) straw (CS), as shown in Figure 7a, the OPC-stabilized clay samples mixed with carbonized straw (CSB) exhibit higher UCS, as shown in Figure 7b,c. The UCS results after 7 days of curing, representing early strength, indicate that all CSB–OPC stabilized clay samples have higher strength than the CS–OPC samples. Moreover, under certain mix proportions, most CSB–OPC samples significantly exceed the strength of the CA sample and exhibit early strength comparable to that of the OPC-stabilized sample. In the 28-day curing tests, representing mid-term strength, the strength gain of the CSB–OPC samples is even more pronounced, and indeed all mix-proportion samples exceed the strength of the CA sample. These results indicate a significant synergistic effect between CSB and OPC in consolidating high-moisture-content clay, suggesting the potential of CSB to replace OPC as a sustainable stabilizer to achieve consistently improved strength in simulated high-water-content dredged clay.

**Figure 8:**
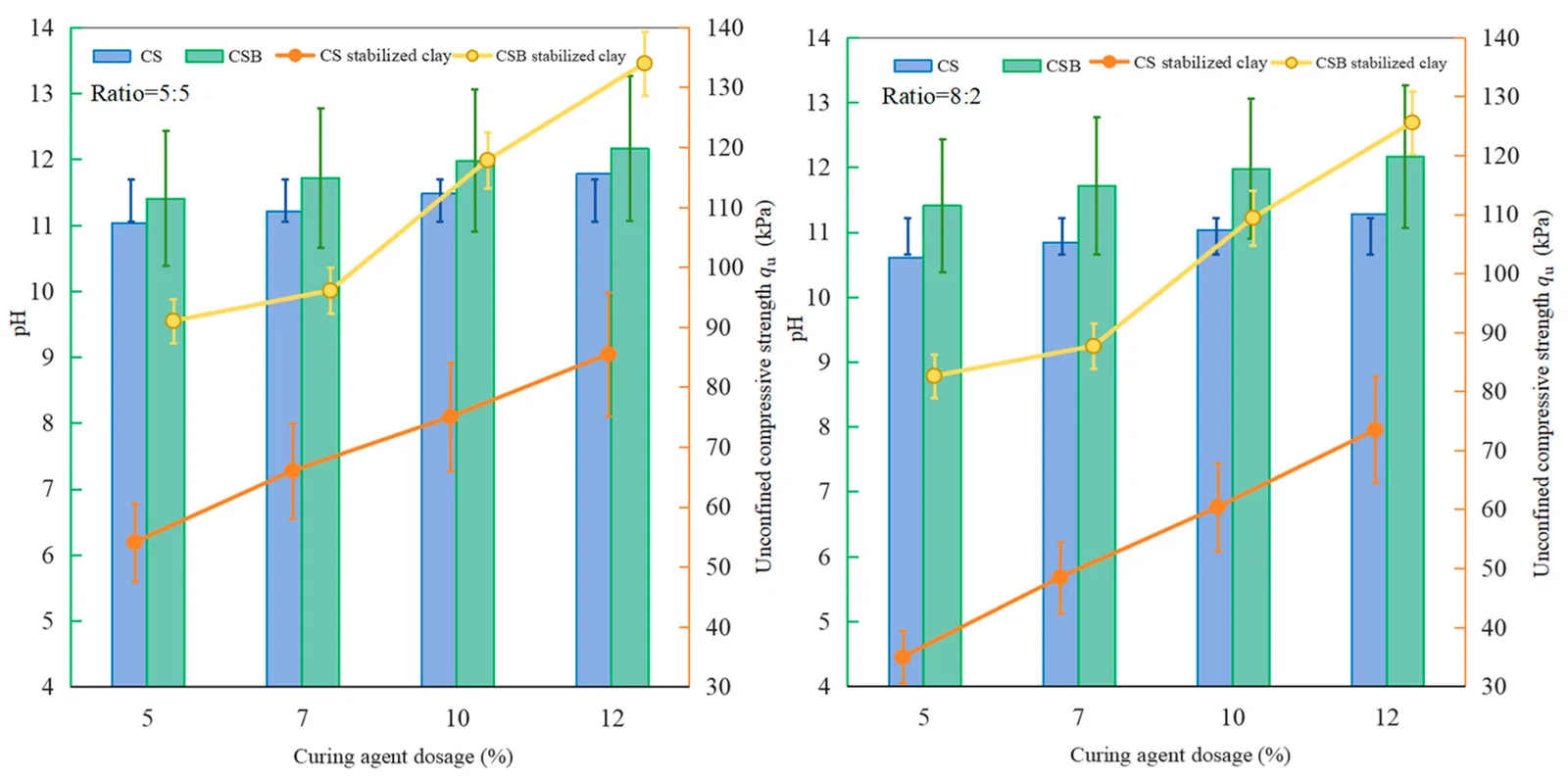
Relationship between pH and UCS of specimens with different ratios after 28 d curing.

Figure 8 shows the relationship between pH and UCS for different samples after 28 days of curing. Both the sample with a higher cement mixing ratio, 5:5, and that with a lower ratio, 2:8, exhibit a positive correlation between pH and UCS. This is because a more alkaline environment is conducive to cement hydration and pozzolanic reactions, which promotes the formation of cementitious products and thus increases sample strength [46]. This indicates that in the CSB samples, high-temperature carbonization has removed some of the organic inhibitory components from the straw, preventing the formation of significant amounts of organic acids, carboxyl groups, and other substances that would weaken the system’s alkaline environment. Compared to the CS samples, the higher strength of the CSB system is not only related to its internal bonding structure, but also to the fact that it can maintain a more favorable alkaline reaction environment, which is another important influencing factor.

### 3.2. Effect of Water Absorption Characteristics of CSB on Strength Development

**Figure 9:**
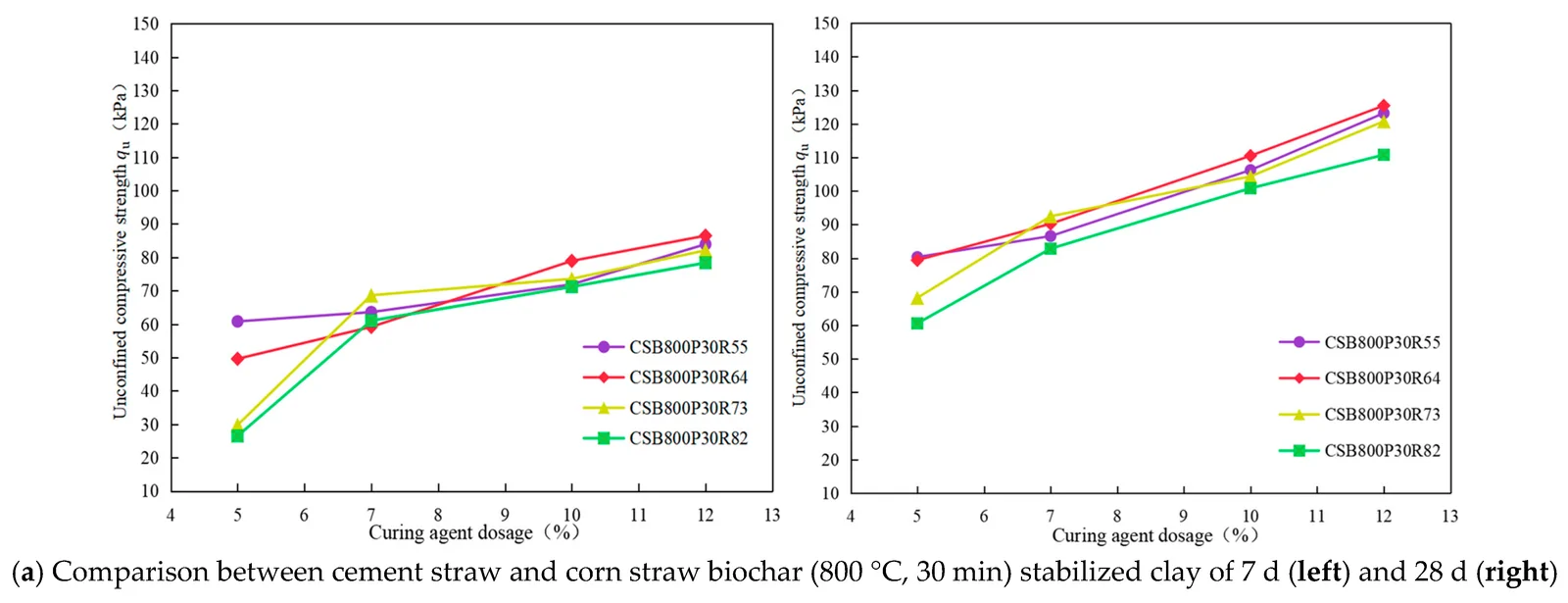

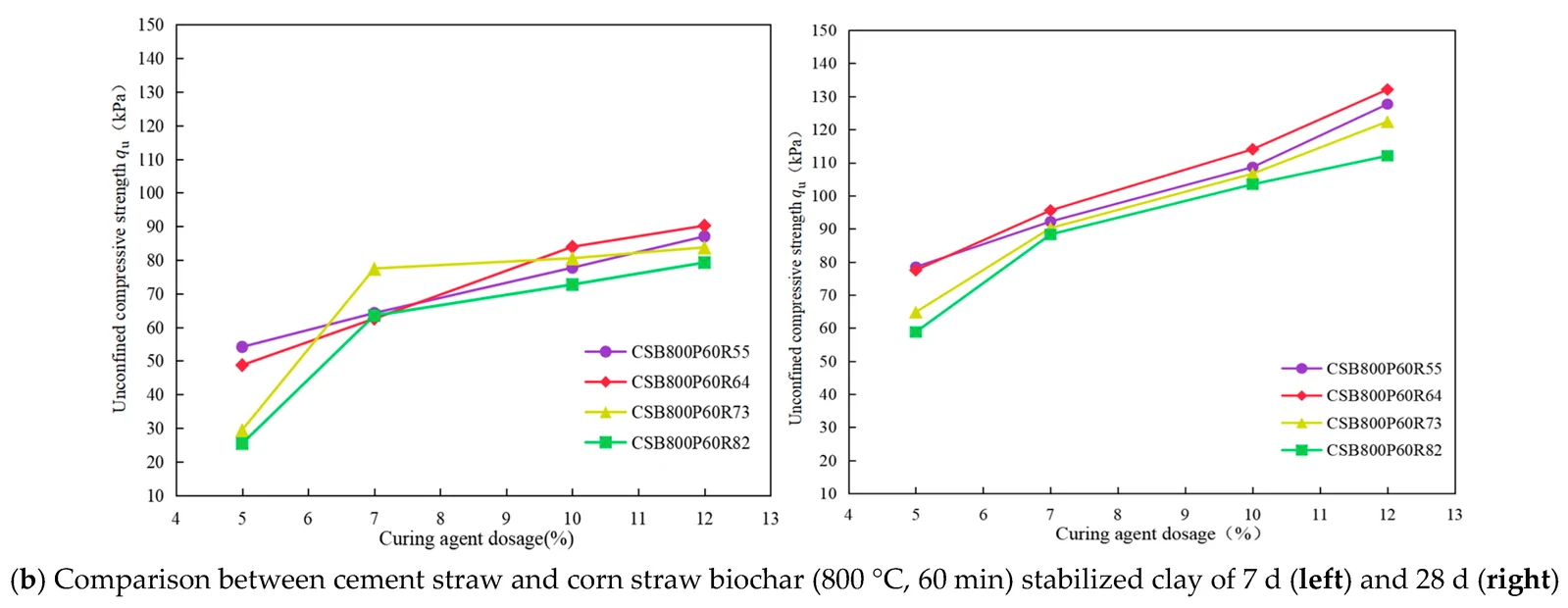
UCS of stabilized clays at different curing agent dosages after 7 d and 28 d of curing.

A comparison of Figure 7b,c shows that the UCS of CSB700P30-stabilized clay specimens was generally higher than that of CSB700P60 specimens. However, when the CSB carbonization temperature increased to 800 °C, the opposite trend was observed, with CSB800P60 specimens exhibiting higher UCS than CSB800P30 specimens, as shown in Figure 9a,b. This indicates that the effect of carbonization duration on strength development is non-monotonic and is closely associated with the evolution of the pore structure and water absorption capacity of CSB.

**Figure 10:**
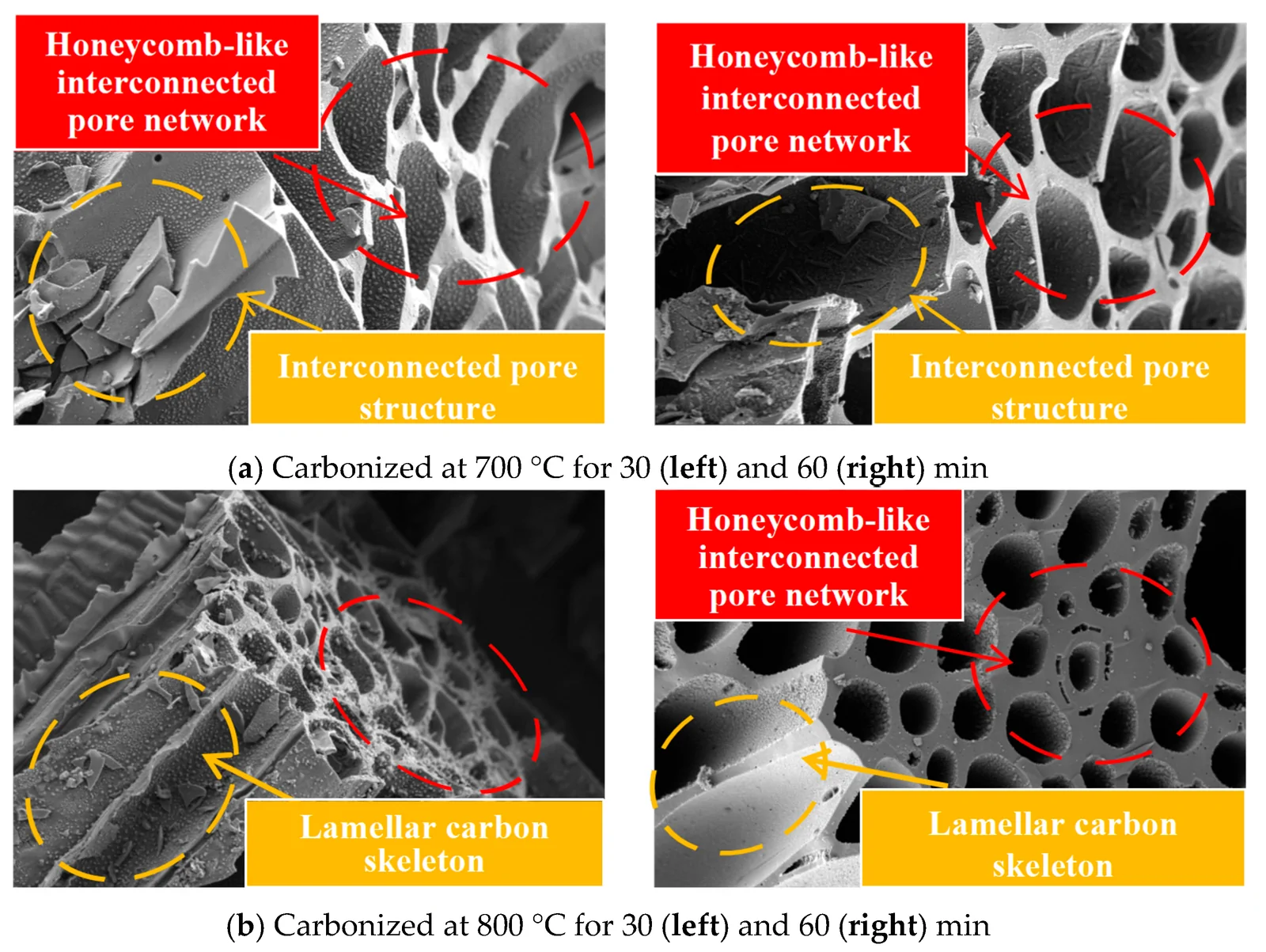
SEM images of corn straw biochar.

Figure 10 further shows the pore structure of CSB prepared under different carbonization conditions. After high-temperature carbonization, the original fibrous tissue disappeared, while the straw skeleton was largely retained. A honeycomb-like interconnected pore structure formed inside the CSB, and a lamellar carbon skeleton developed on the outer surface. Under the P30 condition, CSB carbonized at 700 °C retained a relatively intact cell-wall skeleton, with uniformly distributed micropores and pore channels. When the temperature increased to 800 °C, the carbonization degree increased, but partial shrinkage and collapse of pore walls occurred, reducing the number of micropores and indicating lower water absorption capacity. Under the P60 condition, the cell walls of CSB700P60 became thinner, the pore size increased, and some local pores were damaged, weakening water-storage stability. In contrast, CSB800P60 developed a more pronounced interconnected pore network with a significantly increased number of pores, suggesting higher water absorption capacity. Combined with Figure 4, the UCS variation of stabilized clay specimens was generally consistent with the development trend of CSB water absorption capacity. During hydration, the internal pores of biochar can adsorb and store free water, which is gradually released during curing, thereby promoting cement hydration and the filling of internal pores by cementitious products.

**Figure 11:**
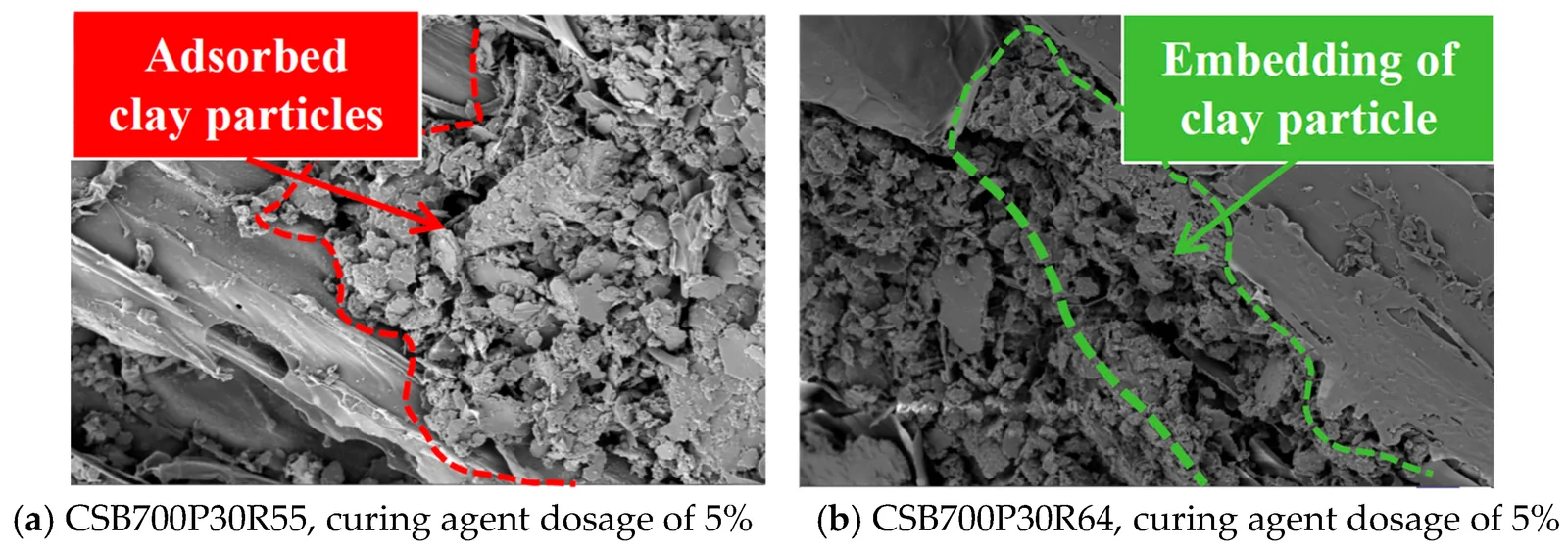

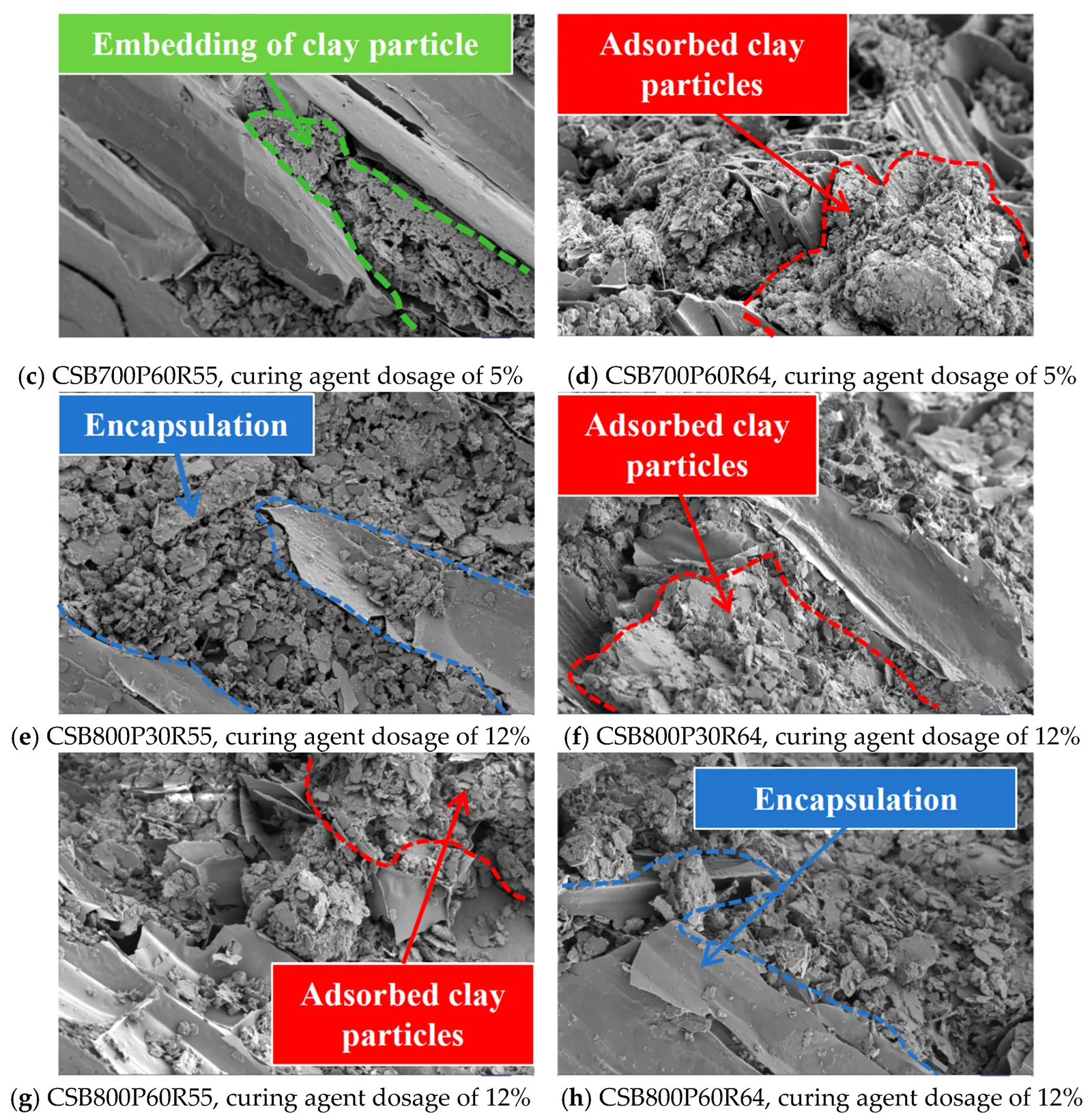
SEM micrographs of high-water-content clay stabilized and modified with corn straw biochar and cement.

**Figure 12:**
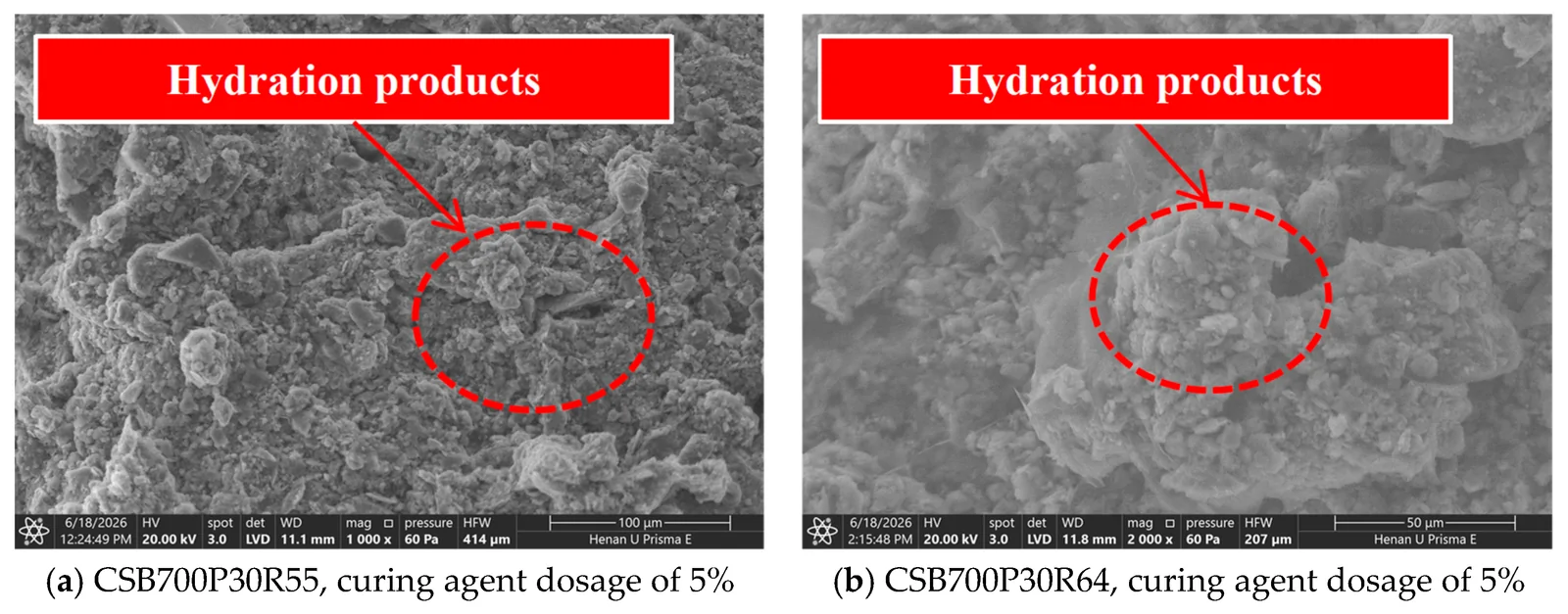

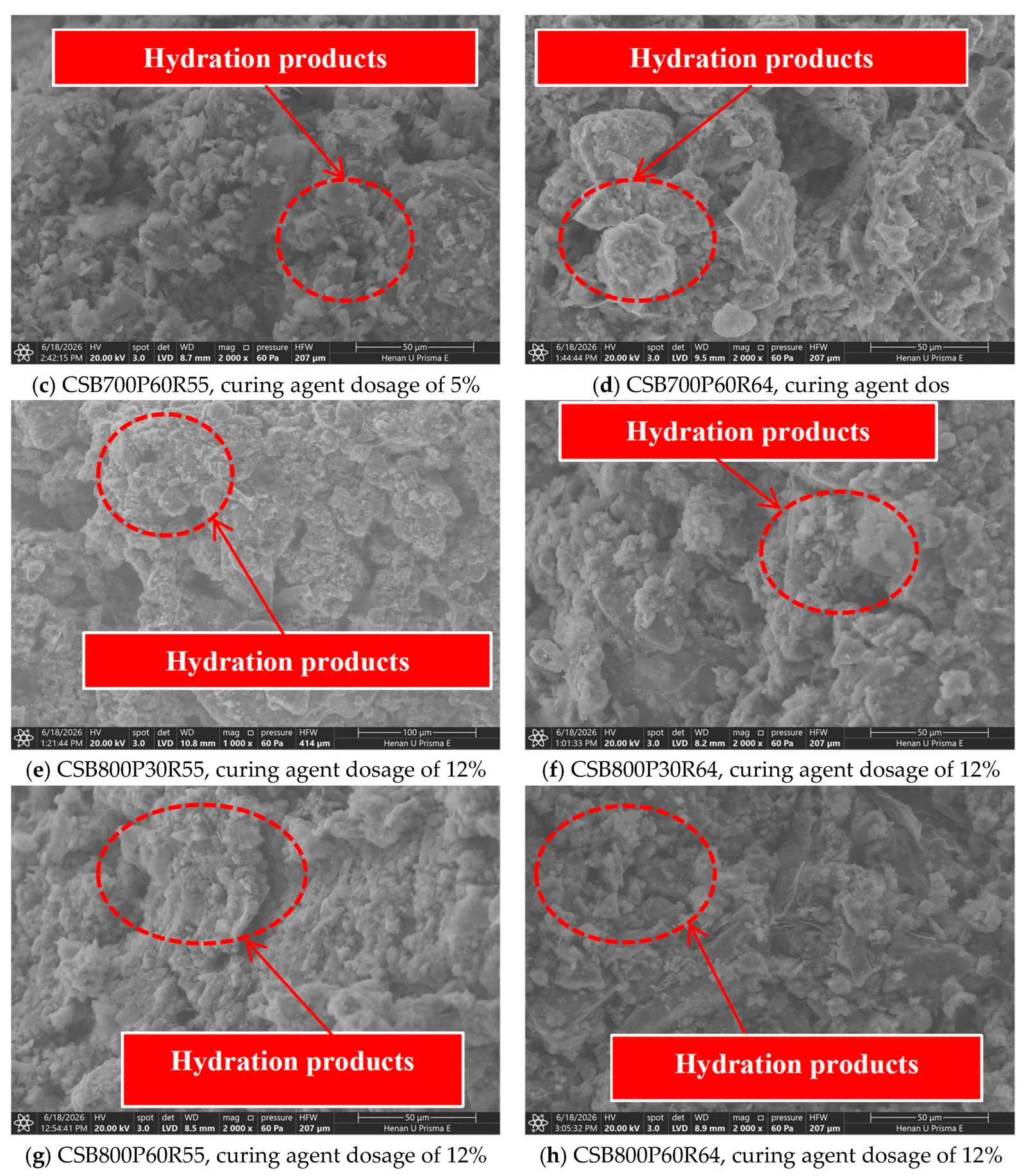
ESEM images of CSB–OPC stabilized clay specimens under different condition.

To further verify the participation of CSB in hydration and clarify its contribution to strength development, field-emission scanning electron microscopy and environmental scanning electron microscopy were performed on CSB–OPC specimens to characterize their dry- and wet-state microstructures, respectively, as shown in Figure 11 and Figure 12. The results show that abundant hydration products and clay particles adhered to the CSB surface, forming embedded, adsorbed, and encapsulated structures. The horizontal comparison in Figure 11 shows that, under the same carbonization condition, specimens with more sufficient particle attachment and pore filling exhibited higher UCS. The specimen with the most distinct encapsulation structure and continuous particle aggregation corresponded to the highest strength. In contrast, CSB700P60R64 was dominated by adsorption, with relatively weak interfacial bonding, resulting in the lowest UCS. The vertical comparison shows that CSB700P30 exhibited more pronounced particle embedding than CSB700P60. However, at 800 °C, extending the carbonization duration to 60 min promoted the formation of a more continuous encapsulation structure, enhanced interfacial cementation and structural compactness, and consequently improved the UCS.

**Figure 13:**
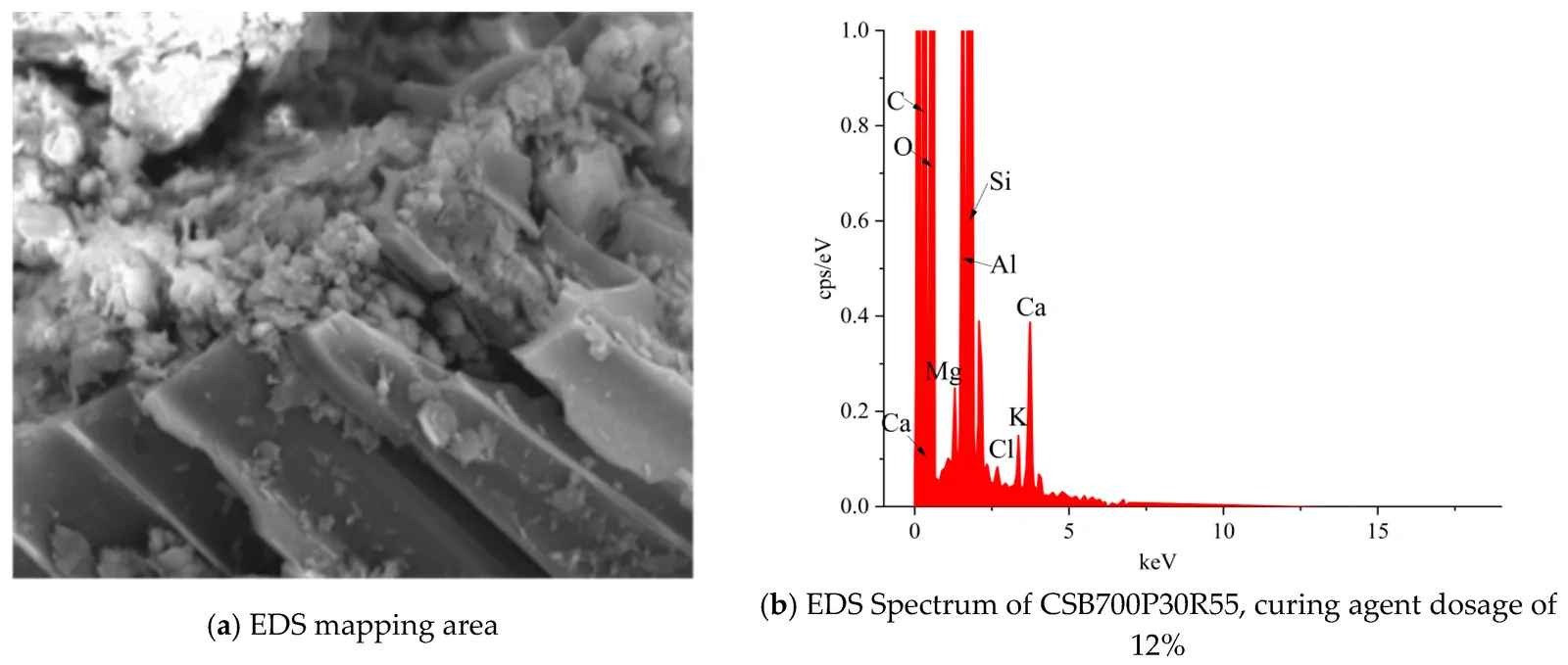

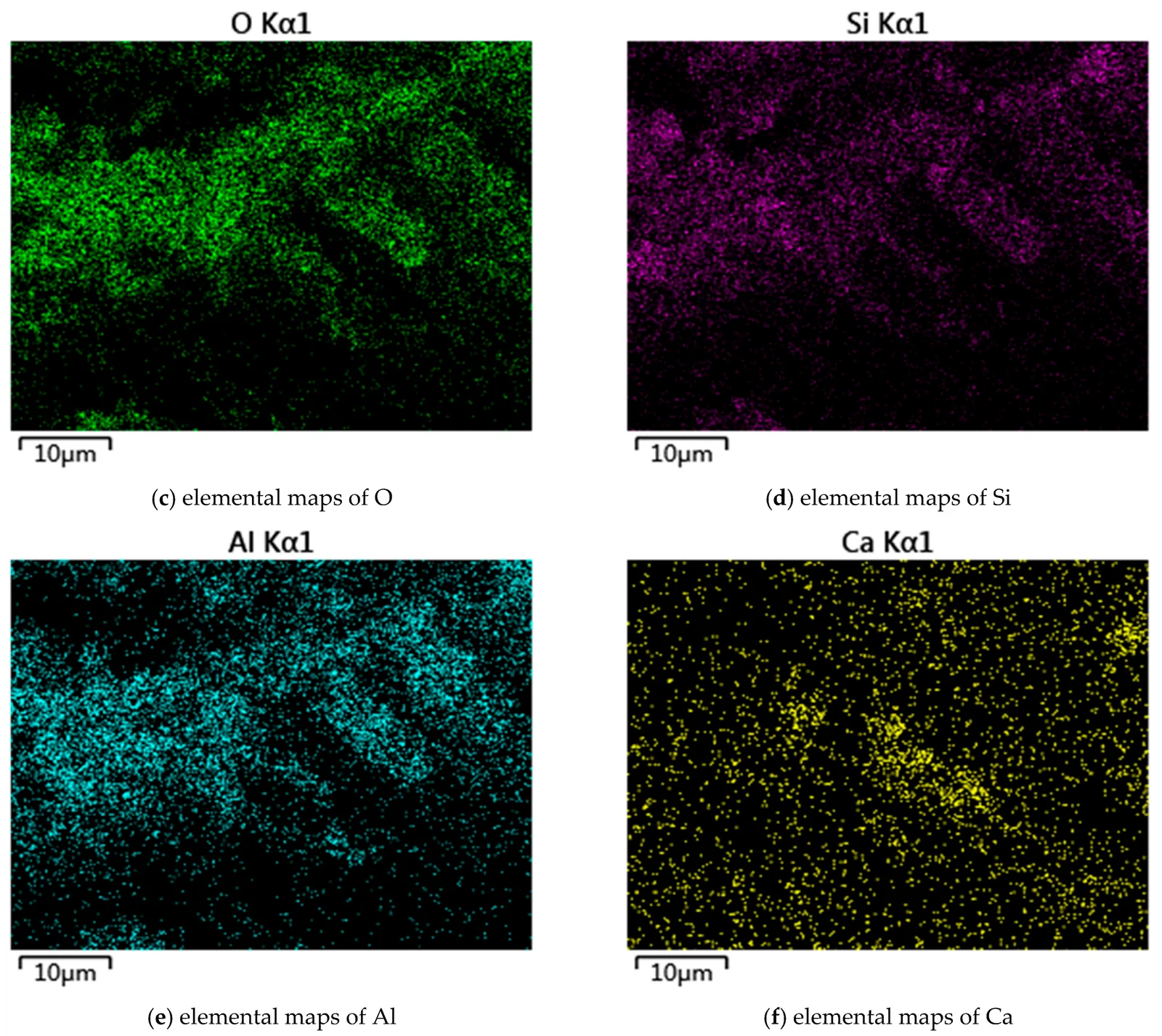
Chemical element compositions of 28-day cured CSB700P30R64 specimens with 12% curing agent dosage measured by EDS (in weight percentage, wt%).

The wet-state microstructures shown in Figure 12 further indicate that high-strength specimens, including CSB800P60R55 and CSB800P60R64, contained abundant flocculent and aggregated cementitious products. These products were widely attached to the surfaces of CSB and clay particles, forming a continuous cementation network. In contrast, fewer hydration products were observed in the low-strength specimens CSB700P60R55 and CSB700P60R64, and the pores were insufficiently filled. This suggests that enhanced hydration promoted the formation of cementitious products and pore filling, thereby improving structural compactness and UCS. EDS spectra and elemental mapping of representative regions, shown in Figure 13, reveal that these regions mainly contained O, Si, Al, and Ca, indicating the presence of Ca-rich hydration products. Combined with the SEM observations, the enrichment of Ca, Si, and O suggests the formation of Ca–Si-rich hydration products, which are likely associated with C–S–H-type gels. These products may contribute to strength development by filling pores and strengthening interparticle cementation.

### 3.3. Influence of Carbonization Temperature on Strength Development of Stabilized Clay

**Figure 14:**
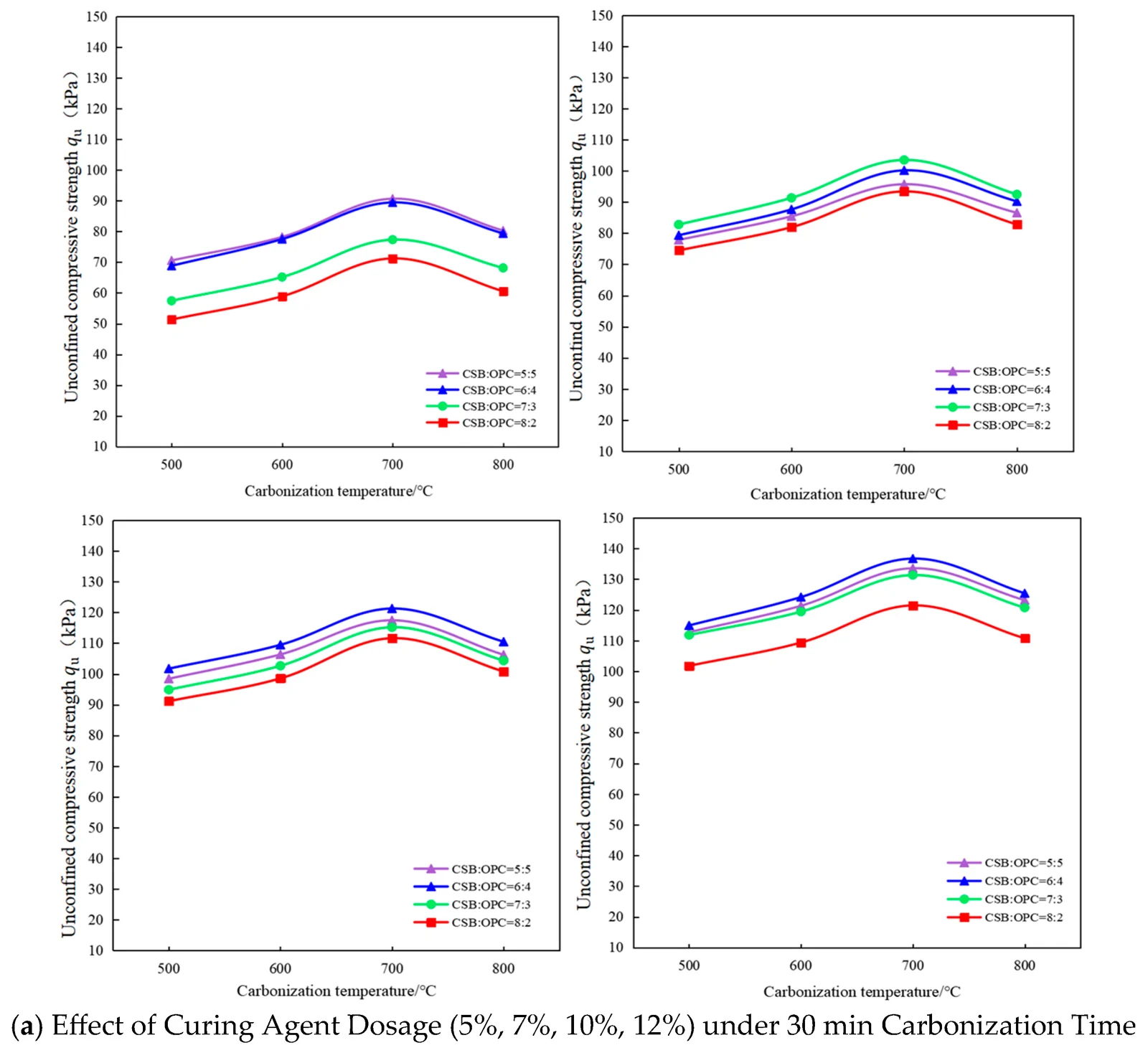

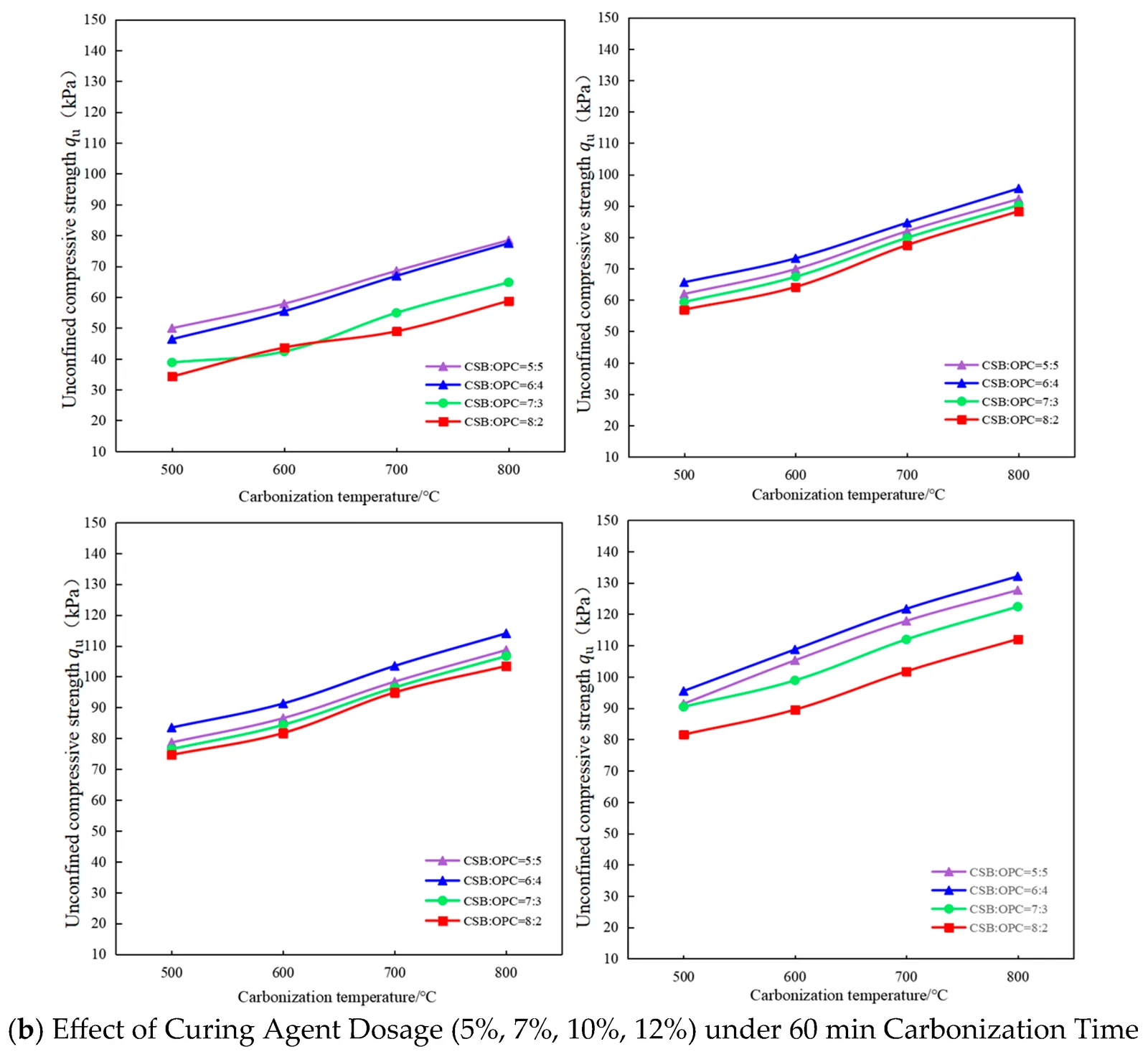
Influence of Carbonization Temperature on the UCS of Stabilized clay.

Figure 14 shows the effect of carbonization temperature on the UCS development of CSB–OPC-stabilized clay under different stabilizer dosages. The UCS evolution differed markedly between the two residence times. For P30 specimens, the UCS generally peaked at 700 °C and slightly decreased at 800 °C, indicating a non-monotonic response to carbonization temperature. In contrast, the UCS of P60 specimens increased continuously within the tested temperature range, with the highest strength mostly obtained at 800 °C. These results suggest that increasing carbonization temperature generally improved the mechanical performance of stabilized clay, although the strength enhancement varied among temperature ranges, indicating a non-linear temperature effect.

The temperature-induced strength enhancement can be mainly attributed to the progressive microstructural evolution of corn straw biochar. As shown in Figure 10, Figure 11 and Figure 12, increasing carbonization temperature promoted the formation of a more distinct hierarchical pore network, a more continuous lamellar carbon skeleton, and a larger specific surface area. Under high-temperature conditions, pore connectivity improved and the honeycomb-like interconnected pore structure became more pronounced, providing favorable pathways for water storage and migration and thereby supporting continuous internal curing during subsequent hydration [47,48]. These structural changes enhanced the water absorption and retention capacity of CSB, enabling sustained water supply for cement hydration. Consequently, cementitious products such as C–S–H gels were continuously generated, filling clay pores and strengthening interparticle bonding. This densified the CSB-modified cement-stabilized clay matrix and ultimately increased the UCS.

The results under different curing conditions further indicate that, within the same carbonization temperature range, the CSB-to-OPC ratio significantly affected the UCS, as shown in Figure 14. In general, the strength increased with increasing cement content. Specimens with CSB ratios of 6:4 and 5:5 exhibited better mechanical performance, whereas those with ratios of 7:3 and 8:2 showed relatively lower strength. This indicates that although CSB can promote hydration through its internal curing effect, excessive cement replacement limits the availability of effective calcium sources required for hydration reactions.

The overall trends under different carbonization conditions further show that increasing temperature improved the strength of all mix-ratio systems, but the degree of enhancement depended strongly on the mix proportion. For the optimal CSB ratios of 6:4 and 5:5, the strength gain induced by increasing temperature was more pronounced, suggesting that sufficient cement hydration products allowed the water storage–release function of CSB to be more fully utilized, forming a denser cemented structure. In contrast, for CSB ratios of 7:3 and 8:2, the limited generation of hydration products restricted pore filling and structural densification, even at higher carbonization temperatures.

Therefore, a clear coupling effect exists between carbonization temperature and mix ratio. Carbonization temperature regulates the internal curing process by controlling the pore structure and adsorption capacity of CSB, whereas the mix ratio determines the upper limit of cementitious product formation. Thus, the strength development of CSB-modified cement-stabilized clay is not governed by a single factor, but by the combined effects of temperature-driven microstructural evolution and mix-ratio-controlled hydration capacity.

## 4. Discussion

### 4.1. Comparison with Previous Studies and Mechanism Interpretation

Previous studies on cement-stabilized dredged or marine clay have shown that UCS generally increases with cement content and curing age, mainly because hydration products fill pores and cement clay particles into a denser matrix. A similar trend was observed in the OPC-only specimens in this study. However, under the high initial water content of 80%, excessive free water may dilute ions, increase diffusion distances, and reduce hydration efficiency. Therefore, increasing OPC dosage can improve strength, but it does not directly address the water-regulation problem and inevitably increases carbon emissions.

The CSB–OPC system showed both similarities to and differences from previously reported biochar-modified cementitious systems. Similar to porous biochar used in cement-based materials, CSB may act as a water reservoir, internal-curing medium, pore-filling modifier, and interfacial attachment site. In this study, the honeycomb-like pores and lamellar carbon skeleton of CSB provided water-storage space, while hydration products and clay particles were adsorbed, embedded, and encapsulated on the CSB surface. Unlike ordinary mortar or concrete systems, however, the present matrix was kaolin-based simulated dredged clay with 80% initial water content; thus, the role of CSB was more closely related to free-water regulation and interfacial bonding under water-rich conditions.

The results also indicate that the effect of CSB was not monotonically improved by increasing carbonization temperature or residence time. CSB700P30 showed the highest water absorption and favorable strength development, whereas longer residence time or higher temperature did not always produce better UCS. This suggests that CSB performance depends on a balance among pore development, carbon skeleton stability, water-retention capacity, and interfacial bonding. It should also be noted that the proposed internal-curing mechanism is indirectly supported by water absorption, UCS, pH, SEM/ESEM, and EDS results. Direct quantification of cement hydration by XRD, FTIR, TG/DTG, or chemically bound water measurements is still needed for further verification.

### 4.2. Mechanical–Environmental Performance and Methodological Assumptions

As described in Section 2.5, a preliminary mechanical–environmental assessment was conducted to compare the carbon-emission efficiency of the OPC-only and CSB–OPC systems. The environmental benefit of CSB–OPC stabilization arises mainly from two aspects. First, partial replacement of OPC by CSB reduces the cement demand required to achieve a given strength level, thereby lowering the carbon-emission intensity of the stabilized clay. Second, CSB is produced from corn straw, an agricultural residue that is otherwise often underutilized or improperly disposed of. Its conversion into a functional stabilizing material links biomass valorization with low-carbon geotechnical treatment.

**Figure 15:**
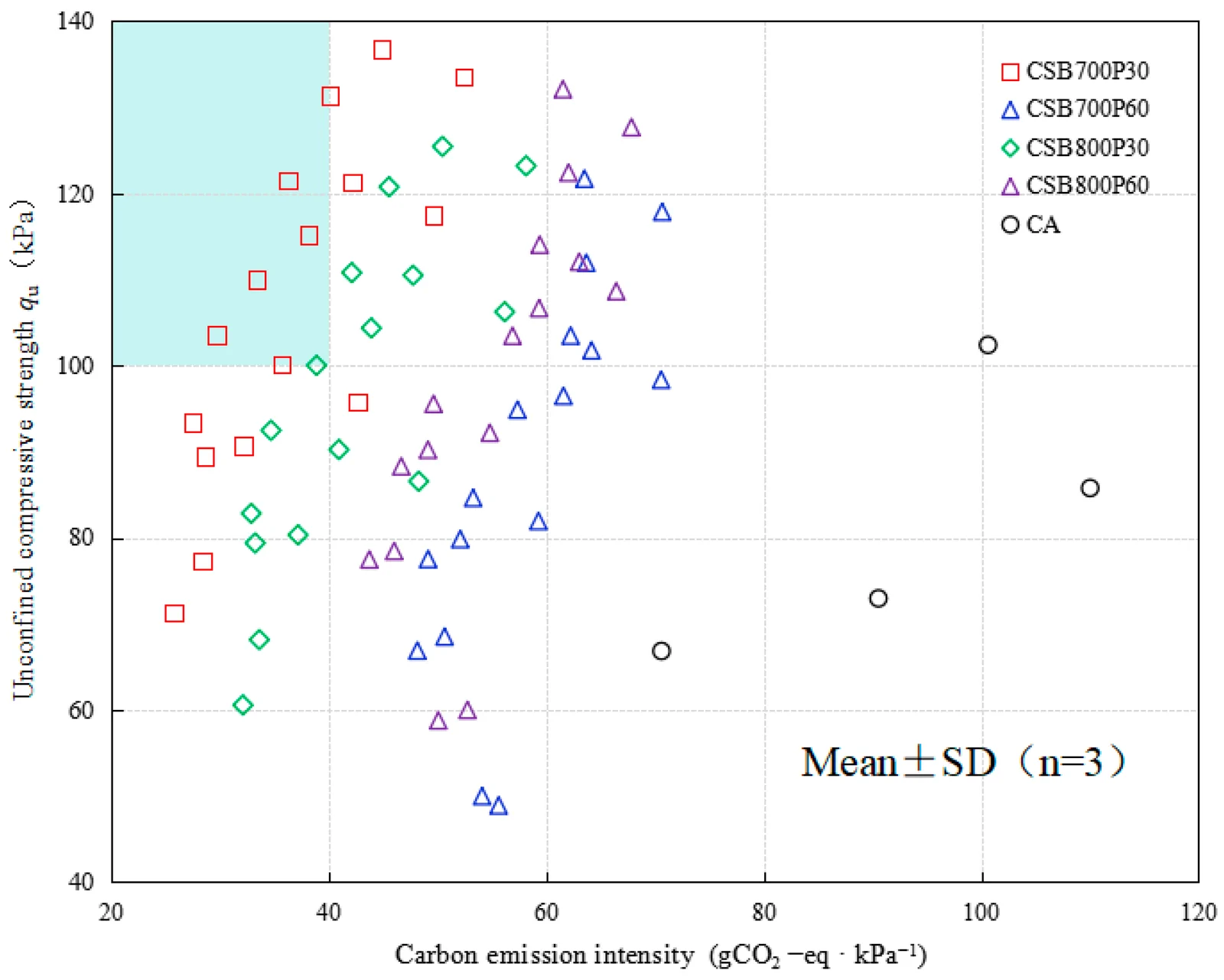
Mechanical–environmental performance of CSB–OPC-stabilized simulated dredged clay.

As shown in Figure 15, Error bars represent the standard deviation of three replicate specimens. Statistical differences among selected mixtures were evaluated by one-way ANOVA followed by Tukey’s test at *p* < 0.05. The CSB–OPC mixtures generally shifted toward the lower-CI region compared with the OPC-only system, indicating that partial OPC replacement by CSB reduced the carbon cost required to achieve a comparable or higher strength level. Among the tested mixtures, CSB700P30 provided the most favorable balance between UCS and CI. This result is consistent with its high water absorption capacity and favorable strength development, suggesting that properly carbonized CSB can improve both mechanical performance and carbon-emission efficiency under the tested conditions.

However, the CI results should be interpreted with caution because the assessment was conducted under a simplified laboratory-scale boundary. Transportation, field mixing, construction equipment, curing management, and end-of-life stages were not included. In addition, the environmental advantage of CSB depends on the balance between OPC reduction and the energy consumed during CSB preparation. Excessive carbonization temperature or residence time may increase preparation energy demand and weaken the overall mechanical–environmental benefit, even when strength improvement is observed in some mixtures. Therefore, the present results should be regarded as a preliminary comparison among the tested binder systems rather than a full life-cycle assessment, and further field-scale life-cycle evaluation is needed before practical application.

### 4.3. Engineering Implications, Limitations, and Future Research

Although the optimum CSB–OPC specimen reached approximately 136–137 kPa at 28 d and exceeded the OPC-only control, the absolute strength level should be critically evaluated. This UCS range indicates improvement in the stability and handling performance of high-water-content clay, but it may not be sufficient for applications requiring high bearing capacity or structural support. Therefore, the proposed CSB–OPC system is more suitable for low- to moderate-strength reuse scenarios, such as land reclamation fill, embankment core materials, backfill, temporary working platforms, and subgrade improvement after additional design verification.

For practical implementation, CSB–OPC stabilization could be incorporated into dredged sediment treatment processes after sediment collection, homogenization, and water-content adjustment. The CSB–OPC binder could then be mixed with dredged clay using in situ or ex situ mixing equipment, followed by curing and quality control based on UCS, water content, pH, and environmental safety indicators. However, the mix design should be adjusted according to the properties of the target sediment, including initial water content, organic matter content, salinity, particle-size distribution, and contaminant composition. Pilot-scale tests are therefore necessary before field application.

Several limitations should be recognized. First, the present work used kaolin-based simulated dredged clay rather than natural dredged sediment. Natural dredged sediments may contain organic matter, soluble salts, sulfides, heavy metals, shell fragments, and complex mineral phases, which can influence cement hydration, CSB–clay interaction, and long-term strength development. Therefore, the conclusions of this study are mainly applicable to the tested simulated clay system and should not be directly generalized to natural dredged sediments.

Second, the characterization of CSB was limited to particle-size distribution, water absorption, thermal behavior, and morphology. Additional parameters such as BET specific surface area, pore-size distribution, elemental composition, ash content, and surface functional groups were not measured, which limits the quantitative interpretation of the differences among biochars prepared under different pyrolysis conditions. Third, long-term durability under wet–dry cycles, freeze–thaw cycles, saline environments, and contaminant exposure was not evaluated in this study. Future work should therefore include field dredged sediments, quantitative hydration analyses, comprehensive CSB characterization, long-term durability tests, leaching assessment, and pilot-scale validation.

Error bars represent the standard deviation of three replicate specimens. Statistical differences among selected mixtures were evaluated by one-way ANOVA followed by Tukey’s test at *p* < 0.05.

## 5. Conclusions

This study investigated the laboratory-scale feasibility of using corn straw biochar (CSB) as a partial OPC substitute for stabilizing kaolin-based simulated high-water-content dredged clay. The main conclusions are as follows:

1. Properly carbonized CSB improved CSB–OPC stabilization compared with untreated straw. The optimum CSB700P30–OPC mixture reached 136–137 kPa at 28 d, exceeding the OPC-only control and suggesting potential for low- to moderate-strength reuse applications.
2. Strength improvement was associated with CSB’s porous structure and high water absorption capacity. CSB700P30 showed the highest water absorption of 1476.10%, while SEM/ESEM observations indicated pore filling, particle attachment, and interfacial bonding around CSB particles.
3. The effect of CSB depended strongly on carbonization temperature and residence time. Higher carbonization severity did not always improve UCS, indicating that strength development requires a balance among pore development, water retention, carbon skeleton stability, and interfacial bonding.
4. The preliminary mechanical–environmental assessment showed that CSB700P30 achieved the best UCS–CI balance among the tested mixtures. However, because simulated clay and simplified environmental assumptions were used, field sediment validation, hydration analysis, durability testing, and pilot-scale assessment remain necessary.

## Figures and Tables

**Table 1:** Physicochemical properties of Kaolin clay.

Properties	Kaolin Clay
Liquid Limit, *w*_L_ (%)	53.26
Plastic Limit, *w*_P_ (%)	33.14
Plastic index, *I*_P_	20.12
Natural water content *w*_n_ (%)	1.89
Density *ρ*_s_ (g/cm^3^)	2.64
Ignition loss LOI (%)	12.58
Fine partical content *F*_c_ (%)	86.70
Coarse particle content *C*_c_ (%)	13.30

**Table 2:** Chemical composition of OPC.

Chemical Compositions	C_3_S	C_2_S	C_3_A	C_4_AF	Gypsum	CaCO_3_	Others
Concentration (%)	54.60	19.60	6.97	9.18	4.59	3.06	2.0

**Table 3:** Preparation of corn straw biochar.

Parameter	Condition/Description
Feedstock	Corn straw stalks
Pre-treatment	Sorted, washed, and cut into 30–50 mm segments
Drying condition	105 °C for 24 h
Sample mass	Approximately 50 g
Pyrolysis container	Sealed metal container, 150 mm inner diameter × 80 mm height
Pyrolysis atmosphere	Static oxygen-limited atmosphere
Heating rate	10 °C/min
Carbonization temperature	200–1000 °C
Residence time	30 and 60 min
Cooling method	Natural cooling to room temperature in the furnace
Post-treatment	Manual grinding and sieving through a 2 mm mesh
Final product	Uniform CSB powder

**Table 4:** Mixing ratio of straw/carbonized straw (700 °C) cement.

Type	Carbonization Time (min)	Curing Agent Dosage (%)	Mixing Ratio of Straw/Carbonized Straw to Cement	Curing Time
Cement	-	5, 7, 10, 12	-	7 d, 28 d
Corn straw	-	5, 7, 10, 12	5:5, 6:4, 7:3, 8:2	
Carbonized corn straw (700 °C)	30			
	60			

**Table 5:** Mixing ratio of straw/carbonized straw (700 °C) cement.

Type	Carbonization Time (min)	Carbonization Temperature (°C)	Curing Agent Dosage (%)	Mixing Ratio of Carbonized Straw to Cement	Curing Time
Carbonized corn straw	30	500	5, 7,10, 12	5:5, 6:4, 7:3, 8:2	28 d
		600			
	60	700			
		800			

**Table 6:** Emission factors and assumptions used for the preliminary mechanical–environmental assessment.

Item	Emission Factor	Unit	Source
OPC production	0.912	g CO_2_-eq/g OPC	Cradle-to-gate emission factor for CEM I Portland cement
Corn straw collection	0	g CO_2_-eq/g dry straw	Treated as agricultural residue; no upstream cultivation burden allocated
Drying electricity	Included in CSB preparation	-	Not separately measured in this study
Pyrolysis energy	Included in CSB preparation	-	Estimated as part of the aggregate CSB preparation burden
Grinding/sieving	Included in CSB preparation	-	Not separately measured in this study
Electricity emission factor	556.8	g CO_2_/kWh	China average electricity emission factor
CSB preparation, total	0.0869	g CO_2_-eq/g CSB	Estimated aggregate value for biochar preparation
Transportation	Excluded	-	Excluded under laboratory-scale material comparison boundary
Field mixing/construction	Excluded	-	Excluded under laboratory-scale material comparison boundary
Curing/end-of-life stage	Excluded	-	Excluded under laboratory-scale material comparison boundary

## Data Availability

The original contributions presented in this study are included in the article. Further inquiries can be directed to the corresponding author.
